# T-DNA characterization of genetically modified 3-R-gene late blight-resistant potato events with a novel procedure utilizing the Samplix Xdrop^®^ enrichment technology

**DOI:** 10.3389/fpls.2024.1330429

**Published:** 2024-02-14

**Authors:** Kelly A. Zarka, Lea Møller Jagd, David S. Douches

**Affiliations:** ^1^ Department of Plant, Soil and Microbial Sciences, Michigan State University, East Lansing, MI, United States; ^2^ Samplix ApS, Birkerød, Denmark

**Keywords:** genetic engineering, insertion site, transformation, flanking sequence, next generation sequencing, potato, *Phytophthora infestans*, T-DNA

## Abstract

Before the commercialization of genetically modified crops, the events carrying the novel DNA must be thoroughly evaluated for agronomic, nutritional, and molecular characteristics. Over the years, polymerase chain reaction-based methods, Southern blot, and short-read sequencing techniques have been utilized for collecting molecular characterization data. Multiple genomic applications are necessary to determine the insert location, flanking sequence analysis, characterization of the inserted DNA, and determination of any interruption of native genes. These techniques are time-consuming and labor-intensive, making it difficult to characterize multiple events. Current advances in sequencing technologies are enabling whole-genomic sequencing of modified crops to obtain full molecular characterization. However, in polyploids, such as the tetraploid potato, it is a challenge to obtain whole-genomic sequencing coverage that meets the regulatory approval of the genetic modification. Here we describe an alternative to labor-intensive applications with a novel procedure using Samplix Xdrop^®^ enrichment technology and next-generation Nanopore sequencing technology to more efficiently characterize the T-DNA insertions of four genetically modified potato events developed by the Feed the Future Global Biotech Potato Partnership: DIA_MSU_UB015, DIA_MSU_UB255, GRA_MSU_UG234, and GRA_MSU_UG265 (derived from regionally important varieties Diamant and Granola). Using the Xdrop® /Nanopore technique, we obtained a very high sequence read coverage within the T-DNA and junction regions. In three of the four events, we were able to use the data to confirm single T-DNA insertions, identify insert locations, identify flanking sequences, and characterize the inserted T-DNA. We further used the characterization data to identify native gene interruption and confirm the stability of the T-DNA across clonal cycles. These results demonstrate the functionality of using the Xdrop® /Nanopore technique for T-DNA characterization. This research will contribute to meeting regulatory safety and regulatory approval requirements for commercialization with small shareholder farmers in target countries within our partnership.

## Introduction

In 2008, the United Nations (UN) declared that it was the “Year of the Potato”. The UN Food and Agriculture Organization (FAO) (2008) stated that “the potato produces more nutritious food, more quickly, on less land, and in harsher climates than any other major crop—up to 85% of the plant is edible human food, compared to around 50% in cereals.” During the past 20 years, there has been a shift toward increased potato production in developing countries in Africa and Asia (FAOSTAT, 2020). Potatoes are high in vitamin C and potassium as well as has a large amount of energy-rich carbohydrates, making them a nutritious food source.

Late blight of potato is a disease caused by *Phytophthora infestans*, and because it can cause drastic reductions in yield, it is recognized as the most important and destructive disease in potatoes worldwide. It is estimated that late blight costs global potato producers over 10 billion USD per year in late blight management and yield losses (Dong and Zhou, 2022). Attempts to provide resistance have been made with genetic modification and conventional breeding. Ghosh et al. (2016) studied the expression of rice oxalate oxidase 4 gene in potato and found that it enhanced late blight resistance. However, how the resistance is achieved is not well understood, and the durability of the resistance has not been studied yet. Conventional breeding, by using wild *Solanum* species that have native genes with resistance (R genes) to late blight, such as *Solanum demissum* (Malcolmson and Black, 1966), has been used to develop high levels of resistance (Nowicki et al., 2012). This resistance is lost quickly in a commercial field environment due to the ability of *P*. *infestans* to evolve and overcome the resistance (Halterman et al., 2010). According to Paluchowska et al. (2022), there have been more than 70 Rpi genes identified and mapped for resistance to strains of *P. infestans*. Nearly 50 of those Rpi genes have been cloned. For the same reasons that conventional breeding fails to produce durable resistance, genetically modifying potatoes with a single R gene has been shown to be overcome by the adaptability of *P. infestans* (Haverkort et al., 2016). Using genetic engineering techniques for stacking of R genes, known to confer a high level of resistance, is a very effective strategy for providing complete resistance to the potato plant and delaying *P. infestans* from overcoming the resistance, as shown by Zhu et al. (2012); Jo et al. (2014); Haesaert et al. (2015), and The Durable Resistance in potato against Phytophthora (DuRPh) project at the Wageningen University (Haverkort et al., 2016). Our collaborators, Ghislain et al. (2019), showed significantly high levels of resistance to *P. infestans* with the stacking of RB and *Rpi-blb2* from *S. bulbocastanum* and *Rpi-vnt1.1* from *S. venturii* in the susceptible potato cultivars Désirée and Victoria when compared to those with a single Rpi gene (RB, *Rpi-blb2* or *Rpi-vnt1.1*).

Feed the Future, the US Government’s global hunger and food security initiative, has made great efforts and enormous strides toward ending global hunger by addressing the main causes of poverty and hunger through partnerships and innovations (Feed the Future Results, 2023). The Feed the Future Global Biotech Potato Partnership is a project funded by the US Agency for International Development (USAID) and implemented by Michigan State University (MSU) in partnership with the International Potato Center (CIP) and leading international organizations, universities, and national research institutions within our target countries of Bangladesh, Indonesia, Kenya, and Nigeria. Our Global Biotech Potato Partnership works to improve food security for small shareholder farmers.

The Global Biotech Potato Partnership project has biotech potato products currently in development, which have three stacked resistance genes from wild potato species and offer broad-spectrum resistance to late blight *P. infestans.* Once the 3-R-gene late blight-resistant potato varieties complete in-country governmental regulatory approval, our project’s stewardship plan will work with target country government institutes and seed industry partners for distribution to smallholder farmers.

Internationally, documents that have been adopted for the guidance for food safety assessments include the Codex Alimentarius Guideline for the Conduct of Food Safety Assessment of Foods Derived from Recombinant-DNA Plants (Codex, 2008) and the Consensus document on the molecular characterization of plants derived from modern biotechnology (OECD, 2010). These guidelines recommend that regulatory packages should include a thorough characterization of the genetically modified (GM) event both molecularly and phenotypically. The characterization data collected usually includes multi-location field trials to compare agronomic/phenotypic characteristics, molecular characterization, and nutritional composition analysis. The results of the GM event are compared to the non-modified plant originally used for the transformation. There is a considerable amount of molecular characterization recommended and Li et al. (2017) recently published a comprehensive review. The recommendations include characterizing the insertion of the T-DNA into the plant from the plasmid. Other recommendations include identifying the number of copies of the T-DNA, determining the intactness of the T-DNA, and identifying the location of the insertion site in the plant genome. Additionally, the flanking region of the insertion in the plant genome should be known so that it can be evaluated for the presence of open reading frames (ORFs). ORFs can indicate a disruption of expression of an existing plant gene or the creation of unintended gene products. These flanking regions are also useful for creating event-specific molecular identity polymerase chain reaction (PCR) assays so that the events created can be confirmed during further analysis and field trials. The Codex guidelines have helped to standardize assessments internationally; however, individual countries have developed their independently established regulatory guidelines. Each of our partner countries has an established government biosafety regulatory framework. A regulatory dossier will be developed for each of our partnerships 3-R-gene late blight-resistant potato events and submitted to each country’s regulatory agency for review and approval. The dossier will include data collection from regulatory field trials, molecular characterization, compositional analyses, environmental impact assessment, and regulatory studies. Molecular characterization of the T-DNA integration into the host genome is a core analysis of the regulatory review process. Molecular characterization is also important for the identification and tracking of transgenic events during the research and commercialization stages. Early characterization of events that include intactness of the T-DNA, the copy number, location of the insertion, and insertion site flanking region sequence analysis also provides insight into the expression of the transgenes.

In the United States, the first genetically modified crop (GM crop) to be approved for commercialization was Calgene’s “Flavr Savr” tomato in 1994 (Kramer and Redenbaugh, 1994). Since then, there have been many methods used for the molecular characterization of T-DNA. Multiple methods are often needed to complete the entire characterization. Southern DNA analysis is an example of a labor-intensive procedure used to analyze T-DNA. For potato products, extensive DNA Southern blot analysis is currently being used. In 2021, the J.R. Simplot Company received a determination of non-regulated status in the United States for their Z6 potato offering late blight protection (single R-gene), low acrylamide potential, lowered reducing sugars, and reduced black spot and browning. Their petition used extensive Southern blot analysis for T-DNA molecular characterization to confirm the copy number and intactness of T-DNA (USDA APHIS, 2021). Different types of PCR methods followed by Sanger sequencing have often been used to identify T-DNA insertion sites and flanking sequences. Ligation-mediated PCR (LM-PCR), inverse PCR (iPCR), and thermal asymmetric interlaced PCR (TAIL-PCR) have been used in addition to Southern blot analysis (Liu et al., 2018; Li et al., 2021). These methods do not often provide complete characterization and are limited due to genomic positions that can be complex due to insertions and deletions. Additionally, these methods are complicated, laborious, and time-consuming.

Advances in sequencing and next-generation sequencing (NGS) have enabled the genomic sequencing of the whole genome. In Pucker et al. (2021), long read sequences were generated with whole-genomic sequencing to characterize T-DNA insertions in *Arabidopsis thaliana*. However, the use of NGS for whole-genome sequencing and characterization of T-DNA in potato, a tetraploid with a haploid length of 840 Mb, is not yet reliable due to the complexity of the genome (Wang et al., 2022). Extensive bioinformatic analysis would be necessary, and further PCR and confirmation sequencing would most likely be necessary to correct gaps. This would be challenging to do when screening large numbers of transformation events. Recently, target capture sequencing (TCS), coupled with Illumina sequencing, was used by collaborators in our partnership to identify and characterize the T-DNA insertion site (Magembe et al., 2023). This method is useful to obtain the flanking regions and insertion sites; however, it was shown that some areas of the T-DNA did not have sufficient coverage. Coverage across the entire T-DNA was not the focus of the paper and was not fully analyzed with the TCS method. Another strategy for T-DNA characterizing involves targeted fragment enrichment called LIFE‐Seq (a universal Large Integrated DNA Fragment Enrichment Sequencing) which has been reported for use in four crops—maize, rice, canola, and soybean (Zhang et al., 2022). This approach utilizes long target DNA enrichment using several applications, including genome DNA random fragmentation, end repair, A-tailing ligation, barcode adapter ligation, size selection, tiling probe hybridization and capture, and long-range PCR. This is similar to the TCS approach in that it uses multiple tiling probes within the T-DNA; however, TCS has smaller sequencing read lengths of 300 bps, whereas Life-Seq uses long-range PCR and obtains much longer sequencing read lengths. Long sequencing reads enable a higher-quality coverage when characterizing large T-DNA regions.

Here we present an alternative target enrichment strategy that we used, in combination with Nanopore sequencing, for the characterization of four transgenic events in potato. An enrichment technology using Xdrop^®^ has been developed by Samplix (Denmark). Xdrop^®^ is described by Samplix as “a versatile and user-friendly instrument for preparing living mammalian or microbial cells, organelles, DNA, or other biological material for high-resolution downstream analyses” (Samplix, 2023). For genomic DNA analysis, Xdrop^®^ can generate single-emulsion droplets for use in workflows, including encapsulating DNA, for unbiased targeted or whole-genome amplification (Samplix, 2023). A detailed overview of the Xdrop^®^ technology is shown in the **Supplementary Material** (**Supplementary Figure S1**), and there is a detailed description in Madsen et al. (2020). Indirect targeted capture, followed by amplification, involves isolating DNA from the organism of interest containing a known sequence of DNA (i.e., T-DNA). The Xdrop^®^ instrument generates droplets and encapsulates DNA fragments. Indirect sequence capture is used followed by multiple displacement amplification to enrich long regions surrounding the known sequence being detected. The enriched DNA can then be sequenced and analyzed (Samplix, 2023). Previously, Samplix described the utilization of Xdrop^®^ for determining T-DNA insert site location (Blondal et al., 2021). The Xdrop^®^ enrichment includes amplification of the targeted T-DNA insert and sequencing. The flanking DNA is obtained *via* physical linkage to the T-DNA sequence. Using long-read sequencing (e.g., PacBio or Oxford Nanopore), it is possible to obtain sequence reads that match some of the T-DNA insert and the flanking genome sequence. This sequence data is then used to determine the genome location of the inserted T-DNA. In this study, the Xdrop^®^ technology was utilized to enrich long fragments that not only allow for the determination of the insert location but also provide high coverage of the entire T-DNA as well as the flanking region. The method described, utilizing the Xdrop^®^ technology and long-read sequence results, provides high-quality data and analysis of T-DNA that includes confirmation of copy number, location of T-DNA insert, sequence of flanking region, and sequence of the entire inserted T-DNA. Analysis of genetically modified crop plants can be more efficiently characterized by using the Xdrop^®^ enrichment technology.

In addition to describing the method for T-DNA characterization, we show how the Xdrop^®^ enrichment technology and next-generation Nanopore sequencing technology are used to characterize the T-DNA insertions for four advanced 3-R-gene late blight-resistant transgenic potato events developed by the Global Biotech Potato Partnership: DIA_MSU_UB015, DIA_MSU_UB255, GRA_MSU_UG234, and GRA_MSU_UG265. The events are in advanced confined field trials and have shown complete (100%) resistance to field late blight strains in Michigan, USA, Indonesia, and Nigeria (Douches, 2021; Douches, 2023). With the sequence data obtained, we describe how these events were then able to be further characterized to identify native gene interruption and confirmation of the stability of the T-DNA across clonal cycles. This molecular analysis will contribute to the regulatory dossier to meet safety and regulatory approval requirements for small shareholder commercialization of our 3-R-gene late blight-resistant potatoes.

## Materials and methods

### Plasmid and T-DNA materials

**Figure 1:**
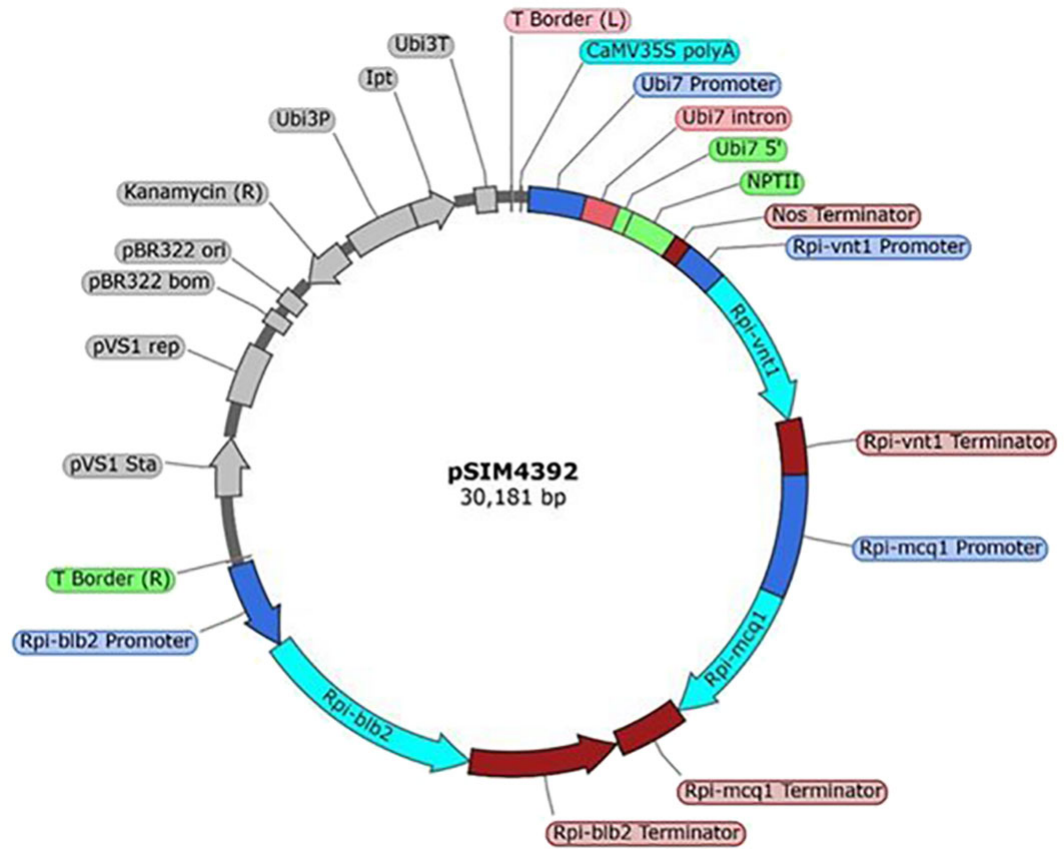
Map of pSIM4392. The T-DNA region extends from coordinates 1 to 21,316 (colored). The backbone region extends from 21,317 to 30,181 (gray). Figure generated using SnapGene software (http://www.snapgene.com/products/snapgene/) (GSL Biotech LLC).

The plasmid pSIM4392 was developed by Simplot Plant Sciences (Boise, ID, USA). The genetic elements within the T-DNA are presented in the **Supplementary Data** (**Supplementary Table S1**). To summarize, pSIM4392 has a T-DNA that contains four cassettes. The first cassette (elements 5 to 11, **Supplementary Table S1**) contains the selectable marker *nptII* gene, and the expression of the gene confers kanamycin resistance used for the selection of plants containing the T-DNA. The second cassette (elements 13–15, **Supplementary Table S1**) contains the *Rpi-vnt1* (*vnt1*) gene from *Solanum venturi* (Foster et al., 2009). The third cassette (elements 17–19, **Supplementary Table S1**) contains the *Rpi-mcq1* (*mcq1*) gene from *Solanum mochiquense* (Aguilera-Galvez et al., 2020). The fourth cassette (elements 21–23, **Supplementary Table S1**) contains the *Rpi-blb2* (*blb2*) gene from *Solanum bulbocastanum* (van der Vossen et al., 2005). The gene products from the last three cassettes—VNT1, MCQ1, and BLB2—are R-proteins involved in the plant immune response that protects potato from foliar late blight infection caused by *P. infestans* (Jones and Dangl, 2006). These genes are in the CC-NB-LRR (coiled-coil, nucleotide-binding, leucine-rich repeat) class of resistance (R) genes (Paluchowska et al. (2022). Each cassette is a cisgene expressed under its native promoter and terminator, pVnt1 and tVnt1 for *Rpi-vnt1*, pMcq1, and tMcq1 for *Rpi-mcq1*, and pBlb2 and tBlb2 for *Rpi-blb2*. The sequence of pSIM4392 plasmid can be found in the Dryad Dataset (Zarka et al., 2023). A map of the entire pSIM4392 plasmid is shown in **Figure 1**.

### Plant materials

Potato plant events were produced using *Agrobacterium* transformation as part of the Global Biotech Potato Partnership and by collaboration with Simplot Plant Sciences (Boise, ID, USA). The C58-derived *Agrobacterium* strain AGL1 (Lazo et al., 1991), carrying pSIM4392, was used to transform potato internode explants following the method described by Richael et al. (2008). A flow chart highlighting the development and selection of lead potato events transformed with T-DNA in plasmid pSIM4392 is shown in the **Supplementary Material** (**Supplementary Figure S2**). The transformed internode explants were regenerated on a medium containing 150 mg/L kanamycin to select for lines containing a T-DNA insert. The pSIM4392 backbone contained the *isopentenyl transferase* (*ipt*) gene. Events expressing the *ipt* gene will have a cytokinin phenotype (stunted growth) or have atypical phenotypes such as elongated trichomes or chlorotic leaves (Kunkel et al., 1999). They would have also transferred some or all of the plasmid backbone. These events were eliminated from further analysis. For both the Diamant and the Granola host varieties, around 300 events were advanced to analyze the T-DNA copy number. The T-DNA copy number was determined by digital droplet polymerase chain reaction (ddPCR) according to the protocol in Collier et al. (2017). Events with more than one copy were eliminated from further analysis. The internal regions of the T-DNA were tested in the events with polymerase chain reaction analysis, and any negative events were eliminated. R-gene function was tested in growth chamber plants’ pathology bioassays and in field trials (Douches, 2021; Douches, 2023). The plant events selected as the lead events and used in this study are DIA_MSU_UB015 and DIA_MSU_UB255 from the host variety Diamant and GRA_MSU_UG234 and GRA_MSU_UG265 from the host variety Granola. These events will be referred to as UB015, UB255, UG234, and UG265, respectively.

### Genomic DNA isolation

For PCR analysis and ddPCR analysis, genomic DNA was isolated from leaf tissue using the DNeasy Plant Mini Kit cat. #69,104 (Qiagen) according to the manufacturer’s instructions. DNA isolation for the Xdrop^®^ enrichment technology method required high-molecular-weight genomic DNA. High-molecular-weight DNA is essential to obtain the long sequencing reads that will span not only the flanking region of the insert location but also the T-DNA. DNA isolation was done with the leaf tissue of greenhouse-grown plants and an isolation procedure modified from Saghai-Maroof et al. (1984). Fresh leaf tissue (2 g) was ground with a mortar and pestle in 7 mL of extraction buffer (0.1 M Tris, pH 8.0/1.4 M NaCl/0.02 M EDTA/2% hexadecyltrimethylammonium bromide/1% 2-mercaptoethanol). Transfer of further DNA-containing solutions was only done with universal pipet tips with wide tip openings (USA Scientific, Ocala, FL, USA). The ground leaf tissue mixture was filtered through two layers of cheesecloth and incubated at 65°C for 30 min with occasional gentle mixing. An equal volume of chloroform/isoamyl alcohol 24:1 (vol:vol) was added, and the solution was mixed by inversion to form an emulsion that was centrifuged at 3,000 rpm for 10 min at room temperature. The aqueous phase was removed, and 2/3 vol of isopropanol was added and mixed by gentle inversions. The precipitated DNA was washed with 1 mL 70% ethanol and then dissolved in 300 μL of resuspension buffer (10 mM Tris and 1 mM EDTA). The DNA samples were evaluated for DNA size distribution by capillary electrophoresis on a Tapestation™ instrument using Genomic DNA ScreenTape (Agilent Inc., Santa Clara, CA, USA) according to the manufacturer’s instructions. The DNA samples were then shipped to Samplix (Denmark).

### Inserted T-DNA analysis

In polyploids, such as the tetraploid potato, it is difficult to have the coverage in whole-genomic sequencing needed to meet regulatory review recommendations. Samplix developed an enrichment instrument technology called the Xdrop^®^, which enables targeted DNA fragments to be encapsulated and enriched so that they can be sequenced using next-generation sequencing. As mentioned in the introduction, Blondal et al. (2021) previously described identifying flanking regions of inserted T-DNA. Here we describe utilizing the technology to achieve high sequence coverage across the entire T-DNA region of each of the lead 3R-gene late blight-resistant events as well as the identification of flanking regions on either side of the T-DNAs.

#### Xdrop^®^ enrichment technology

The Xdrop^®^ enrichment technology uses the Xdrop^®^ instrument, cartridges, and reagents along with the DNA samples of interest. The workflow includes two parts: (1) primer design, enrichment, and quantification and (2) digital polymerase chain reaction (dPCR) generation, sorting of Xdrop^®^ droplets, droplet multiple displacement amplification (dMDA), and evaluation of enrichment. A graphic of the workflow was previously described in Blondal et al. (2021) and included here in the **Supplementary Material** (**Supplementary Figure S1**).

1. Primer design, enrichment, and quantification: The DNA samples were evaluated for size distribution and quality by Tapestation™ System (Agilent Technologies Inc.) using Genomic DNA ScreenTape according to the manufacturer’s instructions. The primer sets for enrichment and quantification were designed specifically for the detection of sequences within the insert site. This was done to achieve coverage across the entire T-DNA and obtain genomic flanking sequence data as well. The primers were tested and successfully implemented to enrich two regions of interest (ROIs) and are listed in the **Supplementary Material** (**Supplementary Table S2**). The primer sets ROI1_8F and ROI1_8R are located within the 3′ region of the *Rpi*-*mcq1* gene. The primer set ROI2_9F and the ROI2_9R set is located within the 3′ region of the *Rpi-vnt1* gene. The highest amount of enrichment will occur in the sequence surrounding the ROIs. Therefore, to ensure that high-quality sequence data can be achieved after the enrichment and span the entire T-DNA, the ROIs are located near the center of the T-DNA. The assay evaluation of primers described in the **Supplementary Material** (**Supplementary Table S2**) was performed by using quantitative polymerase chain reaction (qPCR) as previously described in Blondal et al. (2021).

The DNA samples were purified using HighPrep™ PCR Clean-up Bead System according to the manufacturer’s instructions (MAGBIO Genomics) with the following changes. The bead-to-sample ratios were 1:1 (vol:vol), and elution was performed by heating the sample in the elution buffer for 3 min at 55°C before separation on the magnet. The samples were eluted in 20 μL 10 mM Tris, pH 8. The purified DNA samples were quantified by using Quantus (Promega Inc.) Fluorometer™ according to the manufacturer’s instructions.

2. dPCR generation, sorting of Xdrop^®^ droplets, droplet multiple displacement amplification (dMDA), and evaluation of enrichment: dPCR generation, sorting of Xdrop^®^ droplets, and droplet multiple displacement amplification (dMDA) were performed as previously described in Blondal et al. (2021). In short, millions of double-emulsion droplets were generated by the Xdrop^®^ instrument, followed by droplet PCR (dPCR), which was conducted by taking each DNA sample and compartmentalizing the DNA into droplets that included dPCR master mix and ROI primer sets. After droplet production, the DNA within the droplets were subjected to PCR amplification using the enrichment primers (described above) to generate droplets carrying the ROI. The resulting enrichment of the ROI targets was evaluated by quantitative polymerase chain reaction (qPCR) according to the Xdrop^®^ manufacturer’s instructions. After the dPCR protocol, the droplets were collected and dyed to generate a fluorescent signal in droplets carrying the ROI. The positive droplet populations were sorted from the negative droplets using a fluorescence-activated cell sorting (FACS) instrument, specifically, a SONY benchtop SH800S cell sorter with a 100-μm nozzle (Sony Biotechnology). A more detailed description of the process is presented in the Xdrop^®^ manufacturer’s instructions. DNA from the positive droplets was released and re-encapsulated into single-emulsion droplets by Xdrop^®^ and subjected to multiple displacement amplification (dMDA) according to Xdrop^®^ manufacturer instructions. The amplified DNA was isolated and quantified. The dMDA reactions were then diluted in molecular-grade H_2_O (1:9 vol/vol) and subjected to qPCR reaction using validated qPCR assays. Furthermore, 10 ng of target DNA was used as a control as well as the dMDA controls for background and contamination evaluation. Enrichment Calculation Tool (https://samplix.com/calculations/per-amountof-genetic-material/actual-enrichment-calculator) was used to calculate the enrichment of the dMDA samples according to the Xdrop^®^ manufacturer instructions. Sample cycle threshold (CT) values in real-time polymerase chain reaction (RT-PCR) and the clusters with the FACS analysis were evaluated according to the Xdrop^®^ manufacturer’s instructions.

#### Nanopore genomic sequencing and bioinformatic analysis

Minion Oxford Nanopore Sequencing platform was used to generate long-read sequencing data from the dMDA samples as described by the manufacturer’s instructions (premium whole-genome amplification protocol (SQK-LSK109) with the native barcoding expansions 1–12 and 13–24 (EXP-NBD104/114)). For each of the events, 1.2 μg of amplified dMDA DNA was treated with T7 Endonuclease I, followed by size selection, end-repair, barcoding, and adaptor ligation using the Oxford Nanopore Technology (ONT) Ligation Sequencing Kit. After library generation, the sample was loaded onto a GridION flow cell R9.4.1 (20 fmol) and run for 16–24 hours under standard conditions as recommended by the manufacturer (Oxford Nanopore Inc.). To get sequences with high accuracy, the generated raw data files (fasta5) were base called using Guppy 5.0.17 with super high accuracy and quality 10 filtering.

Using the sequence data obtained and the potato reference genome *Solanum tuberosum* DM1-3 PGSC v4.04 pseudomolecules downloaded from http://spuddb.uga.edu/index.shtml (SPUD DB Potato Genomics Research, 2023), the T-DNA was mapped to the genome with Minimap2. Using the bedtools bamtobed utility, a bed file was created, corresponding to the mapping regions. After masking those regions, using the bedtools maskfasta utility, a new reference genome was created. The T-DNA-only region of the pSIM4392 sequence was then added as an extra chromosome. All reads were then mapped to the new masked genome, containing the T-DNA, using Minimap2 with default settings for Oxford Nanopore reads (-ax map-ont). The sequence viewer Integrative Genomics Viewer (IGV) was used to examine the coverage profile for the T-DNA.

**Figure 2:**
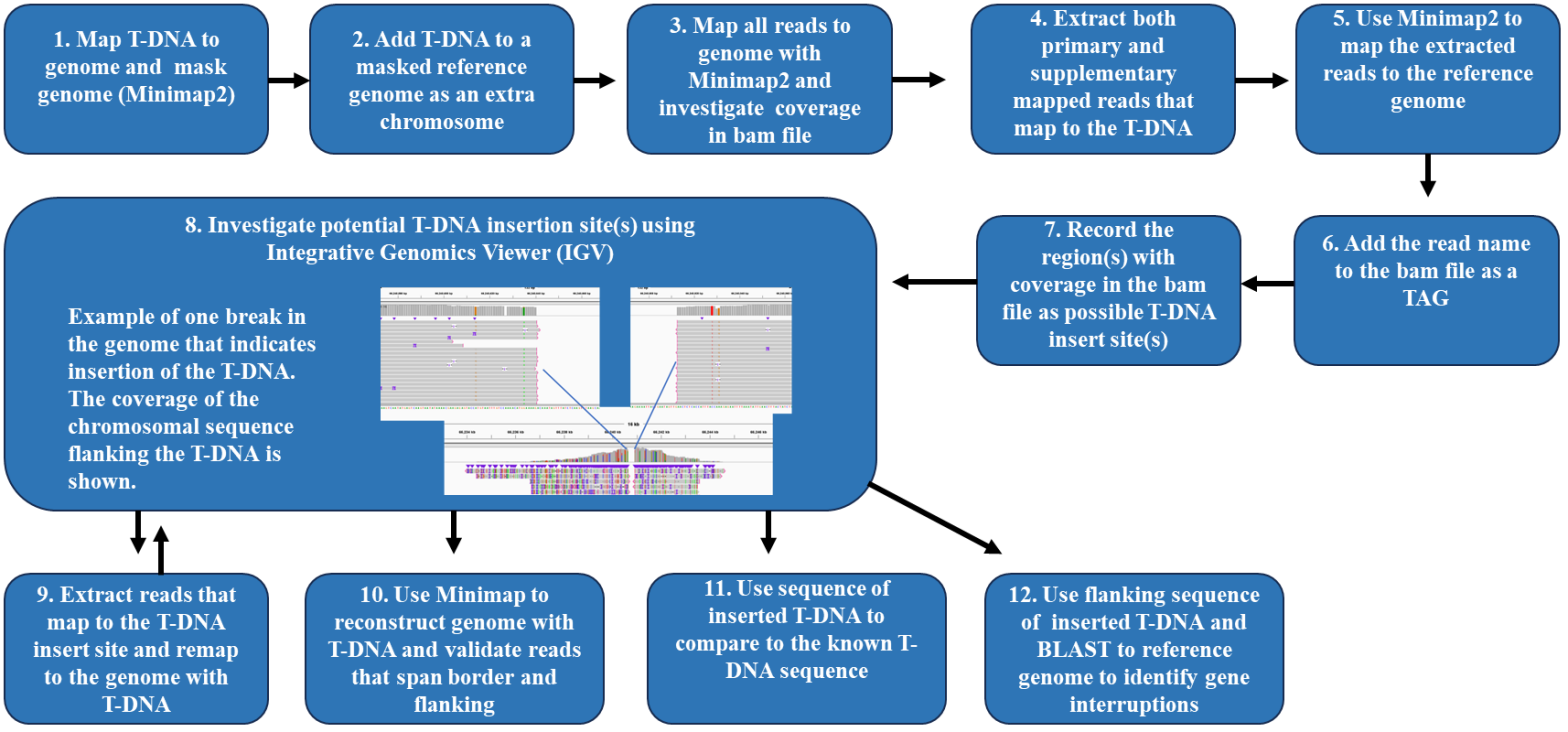
Workflow to characterize T-DNA in a genetically modified event after Xdrop^®^ enrichment and Oxford Nanopore long-read sequencing. The workflow begins with (1) mapping the T-DNA to the genome and masking the genome with Minimap2. (2) The T-DNA is masked to the reference genome and labeled as an extra chromosome. (3) Using Minimap2, all reads are mapped to the genome, and the bam files are investigated for coverage. (4) Both the primary and supplementary mapped reads that map to the T-DNA are extracted. (5) Minimap2 is used to map the extracted reads to the reference genome. (6) The read name is added to the bam file as a TAG. (7) Record the region(s) with coverage in the bam file as possible T-DNA insert site(s). (8) Investigate potential T-DNA insertion sites with the Integrative Genomics Viewer. (9) Reads that map to the T-DNA are extracted and remapped to the genome with the T-DNA. (10) Minimap2 is used to reconstruct the genome with the T-DNA, and reads that span the T-DNA border and flanking regions are validated. (11) The sequence of the inserted T-DNA is compared to the known T-DNA sequence. (12) The flanking sequence of the inserted T-DNA is BLAST-searched to the reference genome to identify gene interruptions.

Both primary and supplementary mapping reads that map to the T-DNA were extracted by using the utilities samtools view and seqkit grep. These are the reads of interest for finding the location of the T-DNA insertions in the genome. Using the reference genome and the extracted reads from the T-DNA, Minimap2 was used to map the reads from the T-DNA to the masked genome which includes the T-DNA. Using the samtools view utility, a TAG containing the read name was added to the bam file, followed by using the bedtools genomecov utility to generate a list of areas with T-DNA coverage in the bam file. The IGV viewer was then used to identify the insertion borders. All sequences from each event were compared with the plasmid T-DNA to determine the integrity of the T-DNA sequences for each event. The workflow for characterizing the T-DNA in a GM event after Xdrop^®^ enrichment and ONT sequencing is described in **Figure 2**. A table of the software, tools, and resources used is available in the **Supplementary Material** (**Supplementary Table S3**).

### Flanking sequence analysis and Sanger sequencing

The reads mapping to the construct were mapped back to the genome to identify the sequence that would span the breakpoint between insert and genome. These sequences were then used to identify the chromosomal location of the insert at each of the left border and the right border T-DNA. The identified insert site was confirmed by PCR across the left and right breakpoints between insert and genome followed by Sanger sequencing. The PCR primers and conditions are found in the **Supplementary Material** (**Supplementary Table S4**). The confirmational PCR analysis and Sanger sequencing covered the junction as well as 1,000 bp of the flanking regions. Sanger sequencing was conducted by submitting amplicon DNA and primers to the Research Technology Support Facility (RTSF) at Michigan State University.

### Insertion analysis in non-GM varieties

The sequence of the potato reference genome *Solanum tuberosum* DM1-3 PGSC v4.04 pseudomolecules downloaded from http://spuddb.uga.edu/index.shtml could be different from the genome sequence of the host varieties of our events. Therefore, to further characterize the insert location, the sequence of the genomic insertion site in the non-GM variety of each event was determined using primers that hybridize to the flanking genomic regions of the insertion sites identified in the flanking sequence analysis. The primers and PCR conditions for this analysis can be found in the **Supplementary Material** (**Supplementary Table S5**). PCR amplification resulted in amplicons that covered the insert loci in the non-GM variety of each event. Each of the resulting fragments were ligated into the TOPO-TA plasmid using Invitrogen™ TOPO™ TA Cloning™ Kit for Sequencing cat. #450030 (according to the manufacturer’s instructions). Each ligation was transformed into Takara’s Stellar™ Competent *E. coli* HST08 strain cells (according to the manufacturer’s instructions). The resulting colonies obtained were PCR-tested using M13 primer sets to confirm the insertion. The selected clones were Sanger-sequenced using the M13 primers. BLAST analysis using the BLAST database (BLAST, 2004) on each of the sequenced amplicons was aligned to a region of the potato reference genome for comparison. The insertion sites for each event were analyzed for chromosomal deletions.

### Native gene interruption analysis

Determination of whether or not a native gene was interrupted by the insertion of the T-DNA in our potato events was studied. The potato reference genome used in this study is from the Potato Genome Sequencing Consortium and is a doubled monoploid (Diambra, 2011). The sequence that they obtained was integrated with a sequence from a heterozygous diploid line and used in the database potato reference genome *Solanum tuberosum* DM1-3 PGSC v4.04 pseudomolecules downloaded from http://spuddb.uga.edu/index.shtml. The insert location on each event was analyzed using the genome browser database within the sequence of the potato reference genome *Solanum tuberosum* DM1-3 PGSC v4.04 pseudomolecules located at http://spuddb.uga.edu/index.shtml to determine if there were any interruption of native genes. The database’s graphical viewer was used to visually inspect the locus for disrupted genes.

### Stability of T-DNA across clonal cycles

Potatoes are propagated asexually and both phenotypically and genetically identical to the mother plant as well as each other. The progeny plants (events) have not recombined meiotically. The stability of the inserts in UB015, UB255, UG234, and UG265 events was assessed to show that DNA introduced into potato through transformation is stable over several clonal cycles. The genetic stability of the T-DNA inserts in UB015, UB255, UG234, and UG265 was assessed using PCR to determine the presence or absence of the insert in plants that have sustained three generations. DNA insert stability was demonstrated in the originally transformed events (G0) by extracting and evaluating DNA from the events that had been propagated *in vitro.* For generation-3 (G3) analyses, three tubers from three plants from each event and a plant from each non-transgenic control were collected from an MSU confined field trial (East Lansing, MI, USA) that sustained three tuber cycles (one in a greenhouse and two in field trials). Genomic DNA from events UB015, UB255, UG234, and UG265 was isolated from fresh plant tissue using Qiagen DNeasy Plant Mini Kit cat no./ID: 69104 according to the manufacturer’s instructions. PCR testing was done with the DNA primer sets found in the **Supplementary Material** (**Supplementary Table S6**). There are two primer sets located within the T-DNA. One set, called VNT1, is located in the native terminator of the *Rpi-vnt1* gene, and the p4274 set is located in the promoter region of the *Rpi-mcq1*gene. The stability of the inserted T-DNA in the events is also shown by PCR analysis using primers that are unique to each event by using a primer set with one primer located in the T-DNA (at either the right or left border) and the other primer located in the chromosomal location specific to the event. There are four sets of T-DNA right border region primer sets, with one set specific for each event. Each PCR was performed using the following conditions: 95°C for 3 min, then 35 cycles at 95°C for 30 s (annealing temp.; see **Supplementary Table S6**)°C 30 s, and 72°C 1 min 20 s. The PCR samples were then electrophoresed on 0.8% agarose gel using a 1-kb standard (STD) from (NEB 1-kb N3232S, New England Biolabs, USA).

## Results

### Copy number results

After transformation, plants that have rooted nicely were selected for copy number analysis. T-DNA copy number was determined by digital droplet polymerase chain reaction (ddPCR) and only the events that contained single copy numbers were used for further analysis. The **Supplementary Data** (**Supplementary Table S7**) shows the results of the ddPCR analysis for the events in this study, with each event containing a single T-DNA insertion. The non-transgenic host varieties Diamant and Granola showed no insert.

### Xdrop^®^ enrichment results

The results of the enrichment are shown in the **Supplementary Material** (**Supplementary Table S8**). Two samples (UG234 and UG265) showed enrichment over 100× as estimated by qPCR, and two samples (UB015 and UB255) had less than 100× enrichment. Since the sample CT values in the RT-PCR were very low and the clusters on the FACS looked very good, the two samples with less than 100× enrichment were passed.

### Sequencing results

The ONT sequencing produced raw data (1.2–1.4 Gb) for each of the samples. The results are shown in the **Supplementary Material** (**Supplementary Table S9**). This amount of raw data provided high-quality coverage of our target regions as described in the intactness of T-DNA and sequence coverage described below.

### Intactness of T-DNA and sequence coverage

The consensus sequences obtained by Xdrop^®^/Nanopore sequencing, from each event, were compared with the pSIM4392 plasmid T-DNA (20,921 bp of T-DNA between the start of the Ubi7 promoter on the left border region and the end of the *Rpi-blb2* promoter on the right border region of the T-DNA) for each event. The consensus sequence for each event matched with the plasmid T-DNA with the following exceptions: at location 368 (of the pSIM4392 plasmid seq.), there is a thymine (T) nucleotide in all of the events tested instead of the expected adenine (A) nucleotide. Prior to this T nucleotide, there is a string of nine T’s followed by an A nucleotide. The T nucleotide appears in all of the events when compared to the plasmid sequence and is located in non-coding DNA. Additionally, there are six nucleotide bases (GCAAGG) located at position 13225 (of the pSIM4392 plasmid seq.). The short segment is deleted in all the events, located in the plasmid T-DNA between the terminator of the *Rpi-mcq* gene and the terminator of *Rpi-blb2* gene and is in non-coding DNA.

The T-DNA insert in each event was further compared to the consensus sequence of the Xdrop^®^/Nanopore sequencing to determine if there were any deletions or insertions of T-DNA at the borders of the insert. Many studies have shown that deletions and insertions often occur during T-DNA transfer into plant chromosomes, and Gelvin (2017) published a review of T-DNA insert integration in plants. **Table 1** shows the results for each of the events. UB255 did not have a T-DNA deletion. UB015 had a deletion of the Ubi7 promoter (originally from potato) that drives the *nptII* antibiotic resistance marker gene in the T-DNA along with some of the Ubi7 intron. Similarly, the UG265 event had a deletion of over half the Ubi7 promoter. The UG234 event had a small deletion of 41 bp of non-functioning DNA at the right border, a 483-bp deletion at the left border region that included part of the Ubi7 promoter, and an insertion of an extra left border region of T-DNA including the Ubi7 promoter and intron (starting 472 bases from the left T-DNA border) inserted into the right border T-DNA in reverse in between the flanking chromosomal DNA and the border. These deletions and insertion of T-DNA did not interfere with the three late blight-resistant genes within the T-DNA, so all events were advanced for further analysis.

**Table 1:** Analysis of deletions and insertions of T-DNA.

Event	T-DNA deletion or insertion	Result of T-DNA deletion or insertion
DIA_MSU_UB015	1,594-bp deletion	Ubi7 promoter driving the *nptII* gene was deleted as well as 360 bp of the Ubi7 intron
DIA_MSU_UB255	None	
GRA_MSU_UG234	483-bp deletion 41-bp deletion 1,067-bp insertion	Left border T-DNA region and part of the Ubi7 promoter Right border T-DNA region An extra left border region of T-DNA including the Ubi7 promoter and intron (starting 472 bases in from left T-DNA border) inserted into the right border T-DNA in reverse in between the flanking chromosomal DNA and the border
GRA_MSU_UG265	889-bp deletion	Left border T-DNA region including over half of the Ubi7 promoter

The table shows a summary describing the deletions or insertions and the result of that deletion or insertion for each potato event.

**Figure 3:**
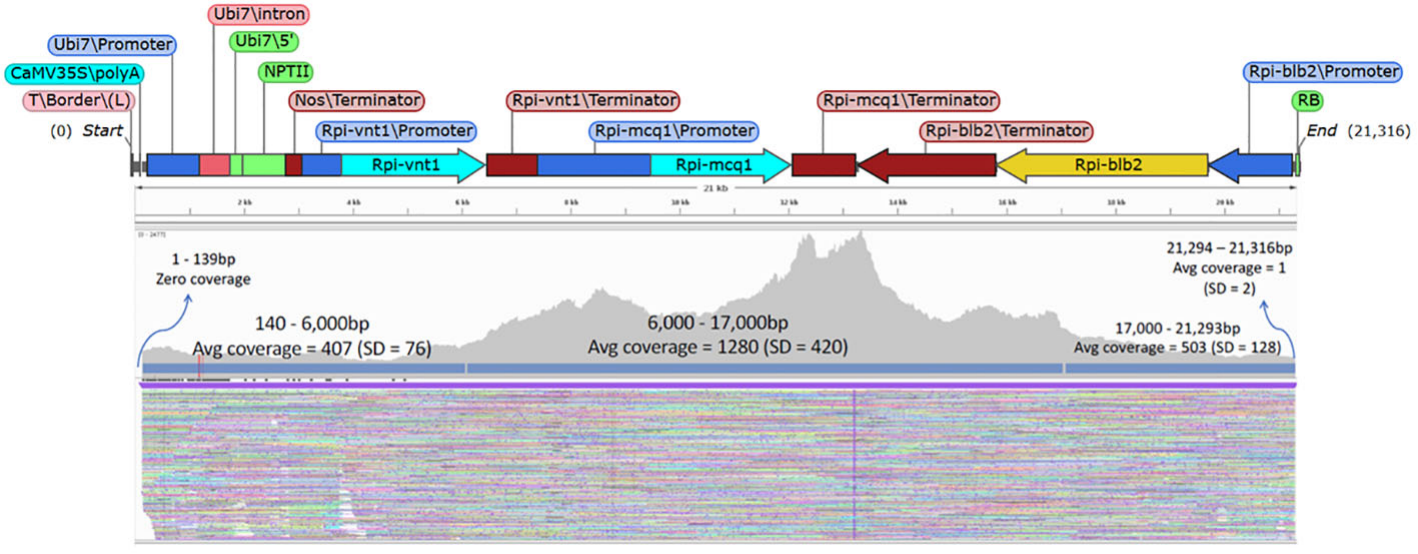
T-DNA coverage of the event MSU_DIA_UB255. The genetic elements of the T-DNA from plasmid pSIM4392 is shown above an Integrative Genomics Viewer image showing the ONT sequencing reads from the event MSU_DIA_UB255. The sequencing reads were mapped to the T-DNA as the reference. There was an average coverage of 1,280× for the center 10 kb of the T-DNA and 400–500× average coverage of the 5-kb region of the T-DNA on the left border and right border.

The goal with the Xdrop^®^/Nanopore sequence was to obtain high-quality sequence coverage across the entire T-DNA insert (the pSIM4392 T-DNA is about 21 kb in size). When the sequence data was mapped to the T-DNA as the reference, the resulting data showed that a very high average coverage can be achieved across the entire region. **Figure 3** shows an example of the coverage obtained with the event UB255 across the T-DNA region. We achieved an average coverage across the center T-DNA (10 kb of the T-DNA) of 1,280× and an average coverage for 5 kb on each side of the T-DNA of 400–500×. The junctions and flanking regions were also covered by the sequencing. **Table 2** shows the coverage achieved in all the events across the T-DNA region.

**Table 2:** Sequencing coverage achieved with Xdrop^®^/Nanopore in the 3-R-gene late blight-resistant events.

Event	Average coverage across the 10-kb center region of the T-DNA	Average coverage across the 5-kb region on each end of the T-DNA
DIA_MSU_UB015	1,200×	300–400×
DIA_MSU_UB255	1,280×	400–500×
GRA_MSU_UG234	1,248×	730×
GRA_MSU_UG265	916×	300–450×

### Insertion location of T-DNA

**Figure 4:**
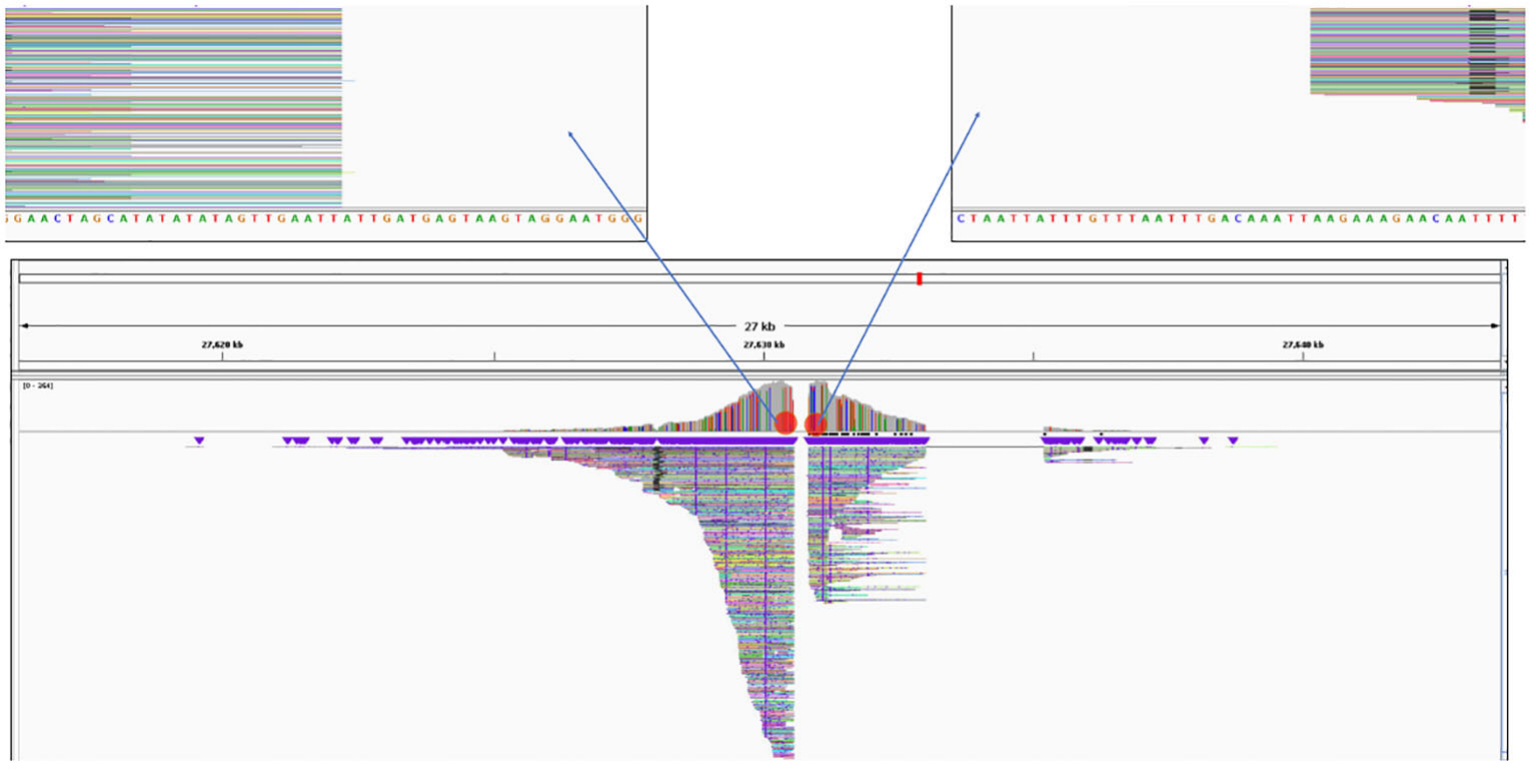
Insertion location of the T-DNA in the event MSU_DIA_UB255 as viewed in Integrative Genomics Viewer (IGV). Image of the insertion location as viewed in IGV with the Oxford Nanopore Technology (ONT) sequencing reads mapped to the potato reference genome *Solanum tuberosum* DM1-3 PGSC v4.04 pseudomolecules. The ONT sequencing reads viewed are the chromosomal regions that flank the T-DNA. The sequences for the flanking region were then identified to have been inserted in chromosome 11 (see “Results”). The blank area between the reads is a short deletion area of the chromosome as described in the results.

The Xdrop^®^/Nanopore sequencing from each of the events was mapped to the potato genome database as described in the bioinformatics methods section. These results were used to determine the insert location of the 3-R-gene late blight-resistant T-DNA in each of the events. In **Figure 4**, the UB255 data exemplifies how the IGV viewer is used to locate where the T-DNA is inserted into the genome. In this event, the T-DNA was determined to be inserted in chromosome 11 with a small 256-bp chromosomal deletion. The sequence of the potato reference genome *Solanum tuberosum* DM1-3 PGSC v4.04 pseudomolecules could be different from the genome sequence of the host varieties of our events. Therefore, to obtain a more accurate characterization of the insert location, the sequence of the genomic insertion site in the non-GM variety of each event was analyzed by PCR, cloning, and Sanger sequencing. The junction and flanking sequence obtained from each of the events in the respective non-GM host variety was analyzed using PCR. Primers that amplified the homologous, native locus in the untransformed Diamant or Granola were used for each event (see methods section “Insertion analysis in non-GM varieties”). The genomic PCR primers that amplify the locus where the T-DNA was inserted in the event UB015 resulted in an approximately 1.4-kb amplicon using the Diamant genomic DNA. The BLAST of the sequenced amplicon in Diamant aligns to a region of the potato reference genome chromosome 1. A comparison of the flanking region sequence obtained with the Xdrop^®^/Nanopore data and the insertion site sequence obtained from the Diamant host amplicon indicated that 275 bp of genomic DNA was deleted as a result of the insertion. The genomic PCR primers that amplify the locus where the T-DNA was inserted in the event UB255 had a PCR amplification resulting in an approximately 1.0-kb amplicon using the Diamant genomic DNA. The BLAST of the sequenced amplicon in Diamant aligns to a region of the potato reference genome chromosome 11. A comparison of the flanking region sequence obtained with the Xdrop^®^/Nanopore data and the insertion site sequence obtained from the Diamant host amplicon indicated that 258 bp of genomic DNA was deleted as a result of the insertion. The genomic PCR primers that amplify the locus where the T-DNA was inserted in the event UG265 had a PCR amplification of an approximately 1.2-kb amplicon using the Granola genomic DNA. The BLAST of the sequenced amplicon in Granola aligns to a region of the potato reference genome chromosome 5. A comparison of the flanking region sequence obtained with the Xdrop^®^/Nanopore data and the insertion site sequence obtained from the Granola host amplicon indicated that 645 bp of genomic DNA was deleted as a result of the insertion. The genomic insertion site in the Granola host was not able to be determined in the event of UG234 due to the insert being located at a highly repetitive region. PCR attempts with candidate flanking regions failed to amplify.

A summary of the chromosome locations of the inserts for each event and any chromosomal deletions that occurred are shown in **Table 3**. The insert location and amount of chromosomal deletion was not able to be determined in the event UG234. The flanking sequence acquired for that event matches 10 different chromosomes, indicating that the insert is located in a highly repetitive region.

**Table 3:** Summary of the location of T-DNA inserts for 3-R-gene late blight-resistant events.

Event	Chromosome	Insert location^a^	Deletion of chromosomal DNA^a^
DIA_MSU_UB015	Chr 1	66240633	66240633–66240907 275-bp deletion
DIA_MSU_UB255	Chr 11	27630548	27630548–27630806 258-bp chromosomal deletion
GRA_MSU_UG234	Unknown	Repetitive flanking region matches 10 chromosomes	Unknown
GRA_MSU_UG265	Chr 5	1082057	1082057–1082702 645-bp chromosomal deletion

a Numbers refer to the location within the designated chromosome in the potato reference genome Solanum tuberosum DM1-3 PGSC v4.04 pseudomolecules downloaded from http://spuddb.uga.edu/index.shtml.

### T-DNA flanking sequence analysis results

The IGV viewer was also used to identify the flanking sequence. **Figure 3** exemplifies the UB255 flanking region data and how it is visualized in IGV. These are the sequencing reads mapped to the potato reference genome *Solanum tuberosum* DM1-3 PGSC v4.04 pseudomolecules, resulting in the identification of the chromosomal flanking region of the inserted T-DNA. To further confirm the junction sequence and flanking region, PCR and Sanger sequencing were completed, as described in the flanking analysis methods section. The results of this analysis are shown in the **Supplementary Data** (**Supplementary Table S10**). As required for regulatory submissions, 1 kb of flanking sequences is included for each event except for UG234, where the sequence was not obtained due to repetitive regions, making PCR amplification for Sanger sequencing difficult. The junction region and 500 bp of the adjacent T-DNA for each of the events were also included.

### Native gene interruption analysis results

**Figure 5:**
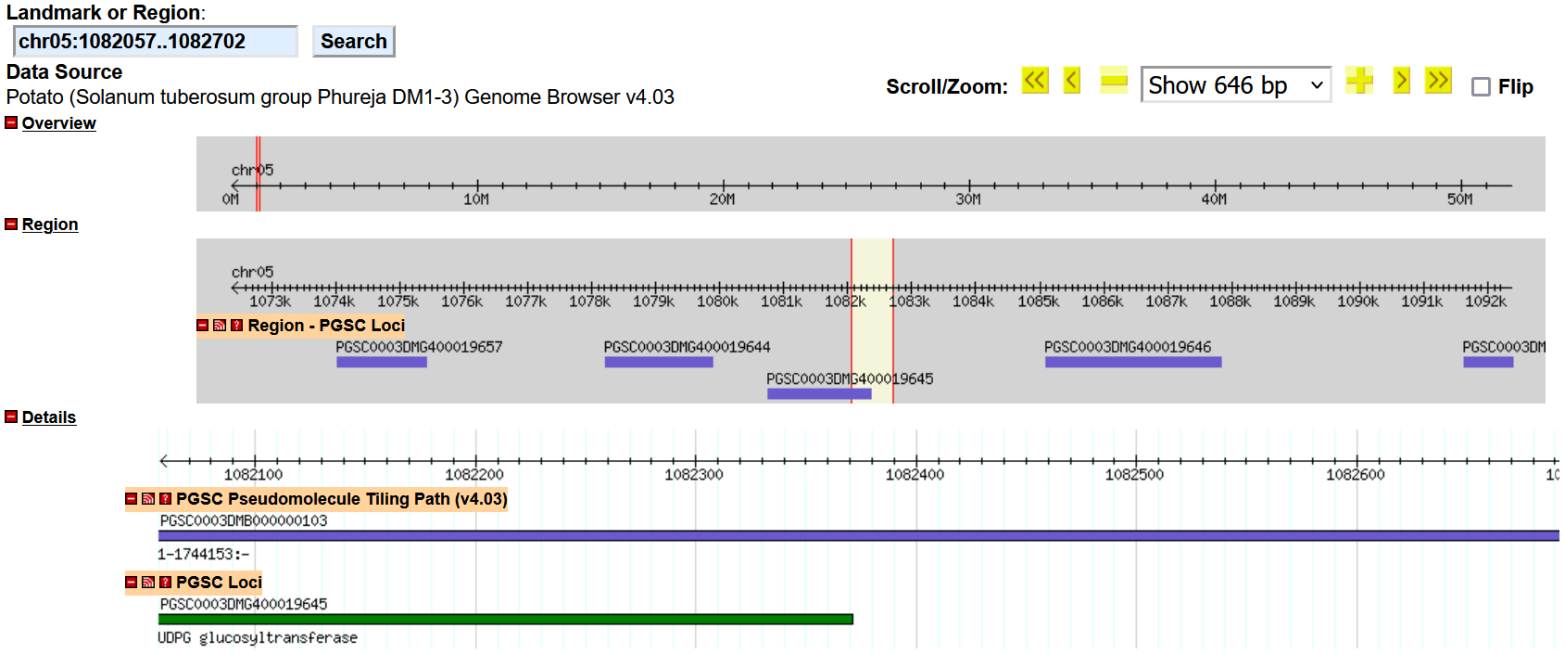
Insert location results for GRA_MSU_UG265 T-DNA. The image above is a graphic from the insert analysis of GRA_MSU_UG265 using the genome browser database within the sequence of the potato reference genome *Solanum tuberosum* DM1-3 PGSC v4.04 pseudomolecules located at http://spuddb.uga.edu/index.shtml. This was used to determine if there were any interruption of native genes. The chromosomal border location of both the right and left borders of the T-DNA insert was inserted into the genome browser. For UG265, the 645-bp region that was deleted from the genome during T-DNA insertion (yellow with red borders) is shown. The insert location in reference to the genome (gray highlight) shows that the gap and insert resulted in a deletion of part of the *UDGP glucosyltransferase* gene at the PCSC loci of PGSC000DMG400019645.

Native gene interruption was studied with each of our events, and both UB015 and UB255 did not show any disruption of genes—see **Supplementary Data** (**Supplementary Figures S3**, **S1**; **Figure 4**, respectively). Due to the repetitive DNA in the flanking chromosomal region of event UG234, interruption analysis could not be performed. Finally, the event UG265 was analyzed, and in the position where the T-DNA was inserted (chromosome 5 at position 1082057–1082702 with a 645-bp deletion; see **Table 3**), there was an interruption and deletion of part of the *UDGP glucosyltransferase* gene (**Figure 5**). Gene interruption may not have a significant impact in tetraploid potato since many of the genes have multiple copies (Sun et al., 2019). The *UDGP glucosyltransferase* gene has three other alleles, and it may not have any functional impact on this event. Further studies, such as expression analysis and compositional analysis, could be done in the future to show that this insertion resulted in no impact.

### Stability of T-DNA across clonal cycles

**Figure 6:**
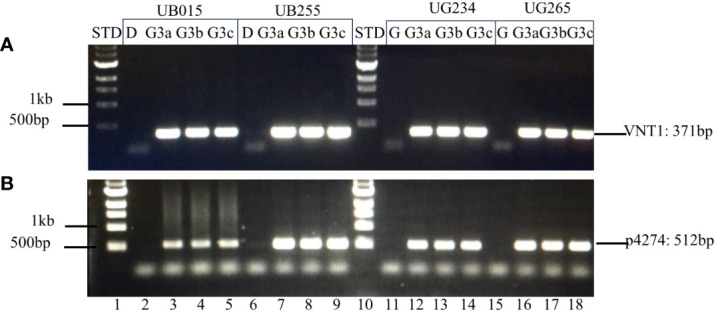
Internal PCR analysis of the T-DNA inserts in G(3) using internal T-DNA primer sets VNT1 and p4274. The stability of the inserted T-DNA in the G(3) of the events is shown by PCR analysis using two internal T-DNA primer sets **(A)** VNT1 with an amplicon of 371 bp and **(B)** p4274 with an amplicon of 512 bp. Lane 1: 1 kb STD (NEB 1 kb N3232S, New England Biolabs, USA); 2–5: Diamant non-transgenic control (D), DIA_MSU_UB015 G(3) tubers a, b, and c respectively; 6–9: Diamant non-transgenic control (D), DIA_MSU_UB255 G(3) tubers a, b, and c respectively; 10: 1 kb STD; 11–14: Granola non-transgenic control (G), GRA_MSU_UG234 G(3) tubers a, b, and c, respectively; 15–18: Granola non-transgenic control (G), GRA_MSU_UG234 G(3) tubers a, b, and c, respectively.

**Figure 7:**
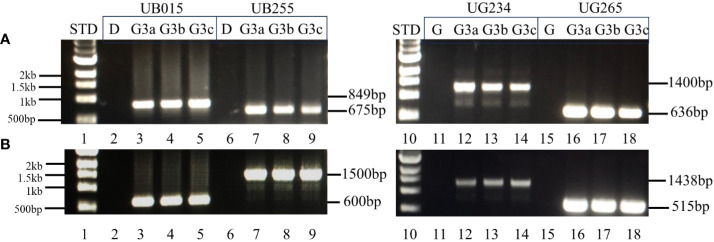
PCR analysis of the T-DNA inserts in G(3) using flanking chromosomal T-DNA primers with primers from the T-DNA in both the left border and right border regions. The stability of the inserted T-DNA in the events is shown by PCR analysis using primers that are specific to each G(3) event by using a primer set with one primer located in the T-DNA (at either the right or left border) and the other primer located in the chromosomal location specific to the event. Segment **(A)** right T-DNA border-specific PCR primer sets. Lane 1: 1 kb STD (NEB 1 kb N3232S, New England Biolabs, USA); 2–5: Diamant non-transgenic control **(D)**, DIA_MSU_UB015 G(3) tubers a, b, and c respectively, amplicon size 849 bp; 6–9: Diamant non-transgenic control (D), DIA_MSU_UB255 G(3) tubers a, b, and c respectively, amplicon size 675 bp; 10: 1 kb STD (NEB 1 kb N3232S, New England Biolabs, USA); 11–14: Granola non-transgenic control (G), GRA_MSU_UG234 G(3) tubers a, b, and c respectively, amplicon size 1,400 bp; 15–18: Granola non-transgenic control (G), GRA_MSU_UG265 G(3) tubers a, b, and c respectively, amplicon size 636 bp. Segment **(B)** left T-DNA border-specific PCR primer sets. Lane 1: 1 kb STD; 2–5: Diamant non-transgenic control (D), DIA_MSU_UB015 G(3) tubers a, b, and c respectively, amplicon size 600 bp; 6–9: Diamant non-transgenic control (D), DIA_MSU_UB255 G(3) tubers a, b, and c respectively, amplicon size 1,500 bp; 10: 1 kb STD; 11–14: Granola non-transgenic control (G), GRA_MSU_UG234 G(3) tubers a, b, and c respectively, amplicon size 1,438 bp; 15–18: Granola non-transgenic control (G), GRA_MSU_UG265 G(3) tubers a, b, and c respectively, amplicon size 515 bp.

The genetic stability of the T-DNA inserts in UB015, UB255, UG234, and UG265 was assessed using PCR to determine the presence or absence of the insert in plants that have sustained three generations. DNA insert stability was demonstrated in the originally transformed events (G0) by extracting and evaluating DNA from the events that had been propagated *in vitro.* For generation-3 (G3) analyses, three tubers (identified as a, b, and c) from three plants from each event were used. The results showed that the T-DNA from events UB015, UB255, UG234, and UG265 is stably inserted into each event. The correct amplicon bands are present in G(0) and G(3) for the two sets of internal PCRs tested. The correct PCR bands are present for the unique sites at both the left and right T-DNA borders in all the events. The results of the G(0) plantlets that have only been in tissue culture are shown in the **Supplementary Material** (**Supplementary Figure S4**). The results of the G(3) for the three tubers a, b, and c with the internal primer sets VNT1 and p4274 are shown in **Figure 6**. The results of the G(3) unique T-DNA left and right border primer sets are shown in **Figure 7**. These results show stability across clonal cycles for all of the 3R-gene late blight-resistant events tested.

## Discussion

In this study, we utilized the Samplix Xdrop^®^ technology to develop a novel procedure to characterize T-DNA more efficiently, and we also characterized our late blight 3-R-gene-resistant potato varieties. We showed that T-DNA copy number, insert location, and T-DNA integrity can be analyzed with no prior knowledge of their genomic integration. Samplix and Blondal et al. (2021) showed that T-DNA flanking regions were able to be determined using Xdrop^®^ when they analyzed a transgenic mouse line. That same method can be used for detecting the integration site and flanking genomic sequence in plants that have integrated T-DNA. We showed that, by isolating high-molecular-weight chromosomal DNA and designing primers, used for enrichment, targeted to the center of T-DNA, downstream Nanopore sequencing results in high-quality sequence coverage across the large 21-kb T-DNA region.

The data and results from this Samplix Xdrop^®^/Nanopore procedure on our late blight-resistant 3-R-gene potato events UB015, UB255, UG234, and UG265 enabled us to confirm a single T-DNA insertion, identify insert location, identify flanking sequence, and characterize the inserted T-DNA. We further used the characterization data to identify native gene interruption and confirm the stability of the T-DNA across clonal cycles. UB255 contained a T-DNA insertion at a single location, and the insert matched the original plasmid T-DNA. The flanking regions were analyzed and showed no deletions or interruptions of native genes. The UB015 event had a T-DNA insertion at a single location and a full deletion of the Ubi7 promoter driving the *nptII* gene. However, the expression of the *nptII* gene has no commercial function in this GM potato. It is a marker gene for antibiotic resistance and is only used during the selection of events after transformation. The UG265 event contained T-DNA inserted at a single location, and the insert matched the original plasmid T-DNA; however, it had a T-DNA deletion of 889 bp that occurred, resulting in a deletion of over half of the Ubi7 promoter driving the *nptII* gene, similar to the T-DNA deletion in the UB015 event. The UG265 event also has a deletion of 645 bp in the chromosome which resulted in a partial deletion of the *UDGP glucosyltransferase* gene. Gene interruption may not have a significant impact in tetraploid potato since many of the genes have multiple copies (Sun et al., 2019). The *UDGP glucosyltransferase* gene has three other alleles, and it may not have any functional impact on this event. Further studies, such as expression analysis and compositional analysis, might be considered if the event advances to regulatory review. Schnell et al. (2015) wrote a review on insertional effects and point to the fact that insertions occur spontaneously and during conventional breeding; therefore, insertions from genetic modification techniques do not result in more risk. Ideally, advanced GM events should not have a T-DNA that interrupts a gene; however, they can still be reviewed.

The analysis of UG234 was the most difficult since the insertion occurred in a very repetitive region. This made sequencing the flanking regions difficult and identifying the insert location impossible. The insert was sequenced and found to match the plasmid T-DNA. However, like UG265, there was a deletion of 483 bp of the T-DNA in the Ubi7 promoter. Additionally, an extra left border region of T-DNA including 1,067 bp of the Ubi7 promoter and intron (starting 472 bases from the left T-DNA border) inserted into the right border T-DNA (in reverse, with the promoter in the direction of the chromosomal DNA), in between the flanking chromosomal DNA and the border. Rearrangements like this are not an unusual occurrence in *Agrobacterium* transfers (Gelvin, 2017; Schouten et al., 2017). Current field studies show that the UG234 event is highly resistant to late blight and is high-yielding. Therefore, further T-DNA characterization and RNA and protein expression analysis might be considered to meet approval recommendations.

The gene interruption observed in the UG265 event and the deletion, insertion, and difficulty in determining the genome insert location observed in the UG234 event are examples of why a more efficient, low-cost, and easy data processing with high accuracy is necessary for T-DNA characterization technologies, like the Xdrop^®^ technology described here. The earlier genetically modified events can be characterized, the earlier decisions can be made on which events to advance in the research and product development pipeline.

There are several key advantages to utilizing the Xdrop^®^ technology for T-DNA characterization. The sequence data obtained offers an all-in-one T-DNA characterization. The enrichment obtained with Xdrop^®^ is compatible with various downstream sequencing platforms including Nanopore (Oxford Nanopore Technologies, Oxford, UK) and PacBio (Menlo Park, CA). There are some disadvantages that might need consideration before utilizing this method. The Xdrop^®^ instrument is versatile and has use in many different applications; however, the cost of the instrument, consumables, and trained labor may be too high for a laboratory that would only utilize the equipment for one application. The necessary training for the instrument might be difficult to maintain in a laboratory that does not have high throughput. Access to the Xdrop^®^ instrument in a local laboratory or the availability of an Xdrop^®^ targeted enrichment service, within a technical research facility, would be cost-effective and efficient for researchers that only require occasional use of the instrument. As described in the introduction, the techniques, target capture sequencing (TCS) coupled with Illumina sequencing (Magembe et al., 2023) and LIFE‐Seq (a universal Large Integrated DNA Fragment Enrichment Sequencing) coupled with PacBio sequencing (Zhang et al., 2022), also offer improved and cost-effective approaches to characterizing T-DNA in genetically modified crops.

This study of the Global Biotech Potato Partnership late blight-resistant 3-R-gene events UB015, UB255, UG234, and UG265 will contribute to the further comprehensive analysis of the events that include plasmid backbone integration analysis, ongoing agronomic field trials, compositional analysis, and gene expression analysis. A combination of these data for the final lead events will be submitted to regulatory agencies to obtain approval for small shareholder commercialization.

## Data availability statement

The data presented in the study are deposited in the Dryad repository, accession number doi: 10.5061/dryad.mcvdnck6h.

## Author contributions

KZ: Conceptualization, Data curation, Formal analysis, Funding acquisition, Methodology, Writing – original draft, Writing – review & editing. LJ: Formal analysis, Methodology, Writing – review & editing. DD: Funding acquisition, Project administration, Supervision, Validation, Writing – review & editing.
