# Supplementary material for: T-DNA characterization of genetically modified 3-R-gene late blight-resistant potato events with a novel procedure utilizing the Samplix Xdrop® enrichment technology
Source: Front Plant Sci. 2024 Feb 14;15:1330429. doi: 10.3389/fpls.2024.1330429 (PMC10900525; doi:10.3389/fpls.2024.1330429)
Supplement: Supplementary file 1 [file DataSheet_1.pdf]

Supplementary Material

T-DNA Characterization of Genetically Modified 3-R-Gene Late Blight Resistant Potato Events with a Novel Procedure Utilizing the Samplix Xdrop® Enrichment Technology

Kelly A. Zarka[1], Lea Møller Jagd[2], David S. Douches[1]

[1]Department of Plant, Soil and Microbial Sciences, Michigan State University, East Lansing, MI, United States, [2]Samplix, Birkerød, Denmark.

*** Correspondence:**
Kelly A. Zarka
zarka@msu.edu

1 Supplementary Figures

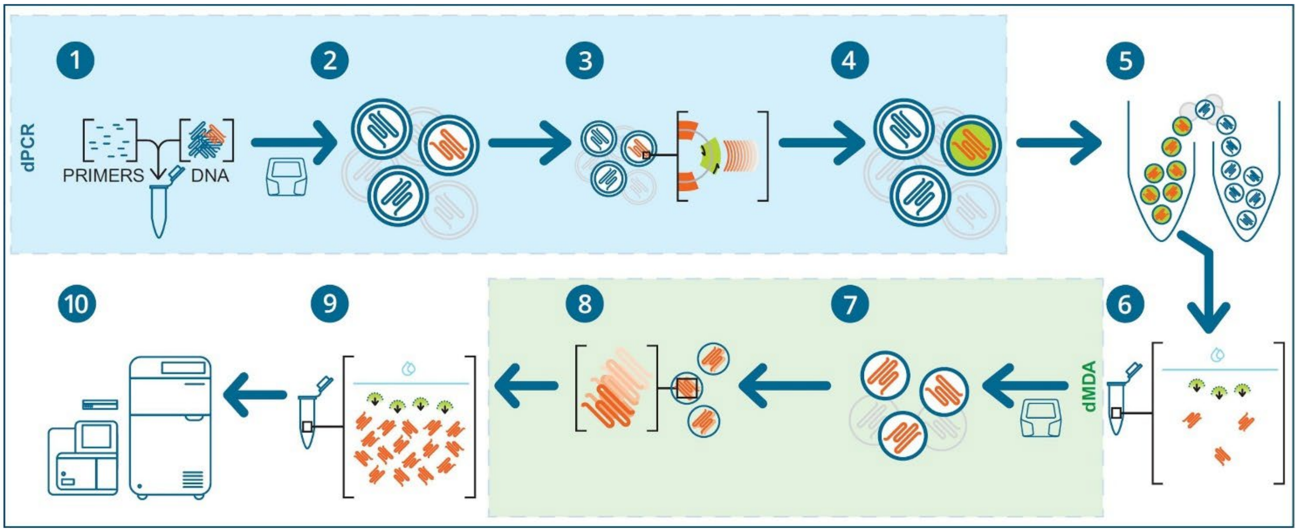

S1 Figure 1. Overview of indirect target sequence capture and unbiased amplification with Xdrop (Samplix, Denmark). The workflow includes indirect sequence capture in double emulsion droplets (dPCR box, blue) and multiple displacement amplification in single emulsion droplets (dMDA box, green). In the first step (1), the sample is mixed with the detection primers and PCR

reagents and this mix (2) is partitioned into millions of double emulsion droplets of ~20 µm diameter using the Xdrop instrument and the dPCR cartridge. These droplets are highly stable and are suitable for standard PCR cycling, flow cytometry analysis, and sorting. (3) Droplets containing the region of interest (ROI) are identified by the detection sequence, a short amplicon (~150 bp) placed within or adjacent to the ROI. Droplets are stained with an intercalating dye (4) and positive droplets are (5) identified by their fluorescence and physically separated from negative droplets using a standard cell sorter. DNA is released from double emulsion droplets (6) resulting in a population of long DNA molecules enriched for the ROI and comprising kilobases of information. For downstream DNA amplification, (7) each long fragment derived from the enrichment is partitioned as single molecules into thousands of single emulsion droplets of ~85 µm diameter for (8) high fidelity multiple displacement amplification in droplets (dMDA). Amplified enriched fragments from the ROI are then (9) released from the single emulsion droplets. The enriched DNA is compatible with (10) downstream analyses, such as long- and short-read sequencing. (Samplix, Denmark)

**1. Assembled and transferred pSIM4392 plasmid into *Agrobacterium tumefaciens* strain AGL1.**
↓
**2. Used *Agrobacterium*-mediated transformation to transform potato internode segments with pSIM4392.**
↓
**3. Promoted callus formation on internode segments using regeneration medium supplemented with 300 mg/l Timentin to inhibit *Agrobacterium* growth and 150 mg/l kanamycin to select for events containing a T-DNA insert.**
↓
**4. Transferred visible shoots to rooting medium containing Timentin and Timentin for continued *Agrobacterium* elimination and kanamycin for selection.**
↓
**5. Plantlets with stunted *ipt*+ phenotype, indicating the presence of pSIM4392 backbone DNA were discarded.**
↓
**6. Two rounds of selective rooting was conducted on plantlets that were growing well and showed rooting in the media. At the same time PCR analysis was conducted to confirm the presence of the T-DNA**
↓
**7. Plants that were PCR positive for the T-DNA from Step 6 were cut and vegetatively propagated for maintenance in tissue culture.**
↓
**8. Tested leaf samples from mature plantlets for the absence of *Agrobacterium* by incubating leaves in NBY broth media for 8 days.**
↓

9. Transferred *Agrobacterium*-free, PCR positive plantlets from tissue culture to greenhouse.

↓

10. Conducted ddPCR analyses to identify events containing single inserts.

↓

11. Pathology assay: grew plantlets in individual pots. Inoculated plantlets with *Phytophthora infestans* spores in a growth chamber and selected events showing no disease symptoms.

S1. Figure 2. Flowchart of the development and selection of lead potato events transformed with T-DNA from plasmid pSIM4392.

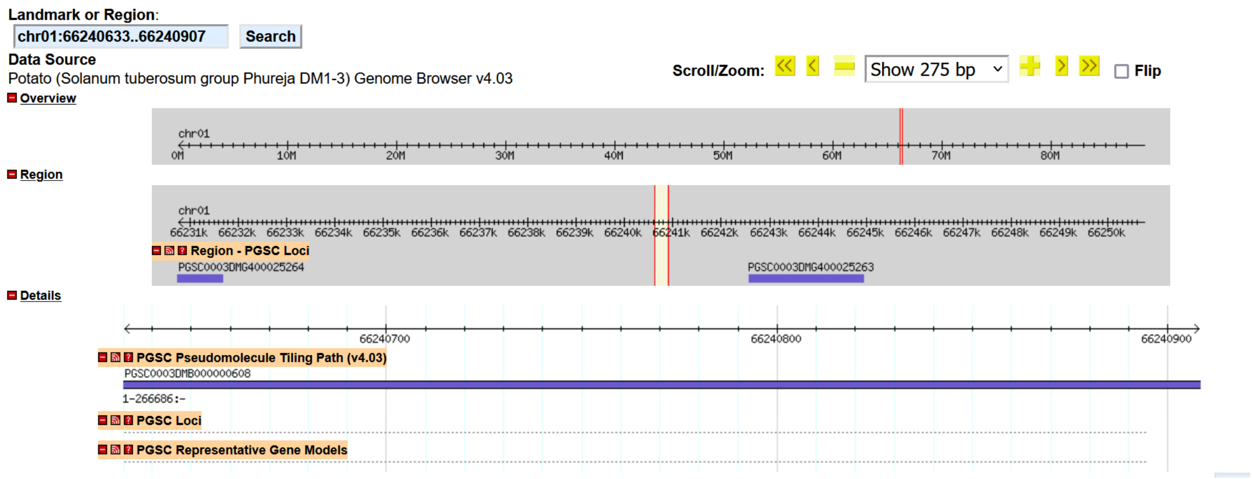

S1. Figure 3. Insert location results for DIA_MSU_UB015 T-DNA. The image above is a graphic from the insert analysis of DIA_MSU_UB015 using the genome browser database within the sequence of the potato reference genome *Solanum tuberosum* DM1-3 PGSC v4.04 pseudomolecules located at http://spuddb.uga.edu/index.shtml. This was used to determine if there were any interruption of native genes. The chromosomal border location of both the right and left border of the T-DNA insert was inserted into the genome browser. For UB015, there is a 275 bp region deleted from the genome during T-DNA insertion (yellow with red borders). The insert location, in reference to the genome (grey highlight), shows that the gap and insert are not interrupting any genes.

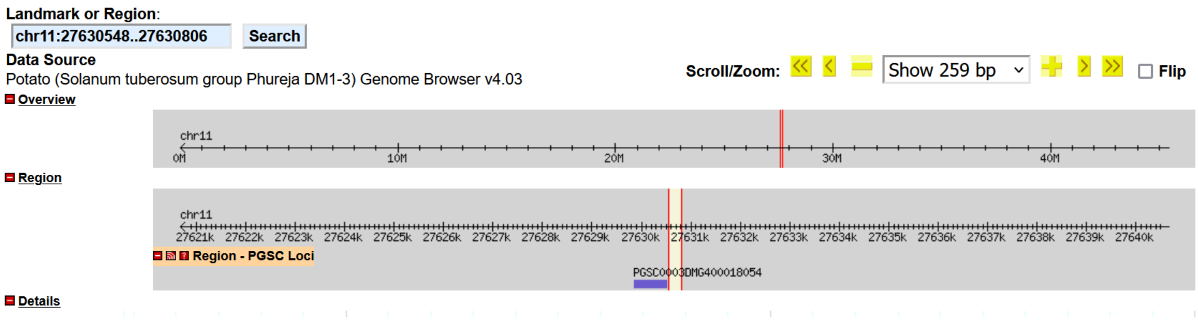

S1. Figure 4. Insert location results for DIA_MSU_UB255 T-DNA. The image above is a graphic from the insert analysis of DIA_MSU_UB255 using the genome browser database within the sequence of the potato reference genome *Solanum tuberosum* DM1-3 PGSC v4.04 pseudomolecules located at http://spuddb.uga.edu/index.shtml. This was used to determine if there were any interruption of native genes. The chromosomal border location of both the right and left border of the T-DNA insert was inserted into the genome browser. For UB255, there is a 258 bp region deleted from the genome during T-DNA insertion (yellow with red borders). The insert location, in reference to the genome (grey highlight), shows that the gap and insert are not interrupting any genes. It was inserted adjacent to the annotation of a putative transcript (purple) PGSC00030DMG400018054.

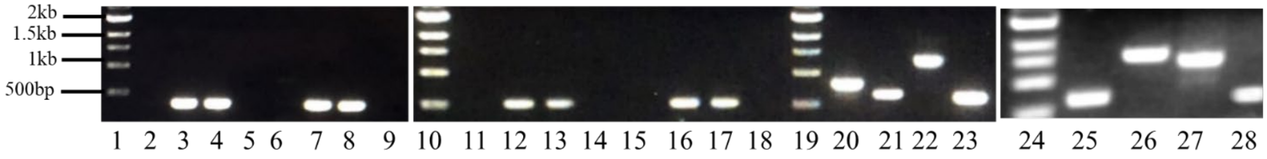

S.1 Figure 5. PCR analysis of the original events G(0) from tissue culture using internal T-DNA (VNT1 gene and p4272) primer sets and primer sets unique to each event. The stability of the T-DNA insert in each event was tested using PCR analysis. This figure shows the results of PCR analysis of the original G(0) events in tissue culture. Lane 1: 1 kb STD, 2-5 VNT1 primer set: Diamant non-transgenic control, DIA_MSU_UB015 G(0), DIA_MSU_UB255 G(0), ddH20, 6-9 VNT primer set. Granola non-transgenic control, GRA_MSU_UG234 G(0), GRA_MSU_UG265 G(0). 10: 1 kb STD, 11-14 p4274 primer set: Diamant non-transgenic control, DIA_MSU_UB015 G(0), DIA_MSU_UB255 G(0), ddH20, 15-18 p4274 primer set: Granola non-transgenic control, GRA_MSU_UG234 G(0), GRA_MSU_UG265 G(0). Lane 19: 1 kb STD, 20-23 Right unique primer set: DIA_MSU_UB015 G(0), DIA_MSU_UB255 G(0) GRA_MSU_UG234 G(0), GRA_MSU_UG265 G(0). 24: 1 kb STD, Left unique primer set: 25-28 DIA_MSU_UB015 G(0), DIA_MSU_UB255 G(0), GRA_MSU_UG234 G(0), GRA_MSU_UG265 G(0).

2 Supplementary Tables

S2. Table 1. Genetic Elements of pSIM4392 T-DNA. pSIM4392 has a T-DNA that contains four cassettes. The first cassette (elements 5 to 11, in yellow) contains the selectable marker *nptII* gene and the expression of the gene confers kanamycin resistance used for the selection of plants containing the T-DNA. The second cassette (elements 13-15, in blue) contains *Rpi-vnt1 (vnt1)* gene from *Solanum venturi*. The third cassette (elements 17-19, in purple) contains *Rpi-mcq1 (mcq1)* gene from *Solanum mochiquense*. The fourth cassette (elements 21-23, in green) contains *Rpi-blb2 (blb2)* gene from *Solanum bulbocastanum*. The gene products from the last three cassettes, VNT1, MCQ1 and BLB2, are R-proteins involved in the plant immune response that protects potato from foliar late blight infection caused by *P. infestans*.

2 Genetic Element (Plasmid Map Label)	Origin	Accession Number[1]	Position (pSIM1678)	Size (bp)	Intended Function
1. Left Border (LB) region	*A. tumefaciens* Ti-plasmid	AF234297	1–26	26	Secondary cleavage site releases ssDNA insert from pSIM4392
2. Intervening sequence	Synthetic	AF234297	27-92	66	Sequence from pCambia1301
3. CaMV poly(A) signal	*Cauliflower mosaic virus*	AF234297	93-282	190	Sequence from pCambia1301
4. Intervening sequence	Synthetic		283-291	9	Sequence used for DNA cloning
5. *StUbi7* gene promoter	*S. tuberosum*	HM439286	292 - 1,234	943	Drives expression of the *nptII* gene
6. *StUbi7* gene intron	*S. tuberosum*	HM439286	1,235 - 1,803	569	Improves expression of *nptII*
7. *StUbi7* gene monomer	*S. tuberosum*	HM439286	1,804 – 2,031	228	Expressed as a fusion with *nptII*, then cleaved off
8. Intervening sequence	Synthetic		2,032 – 2,037	6	Sequence used for DNA cloning
9. NptII	*E. coli*		2,038 – 2,832	795	Confers kanamycin resistance in plants
10. Intervening sequence	Synthetic		2,833 – 2,838	6	Sequence used for DNA cloning
11. Nos	*A. tumefaciens*		2,839 – 3,114	276	Terminates transcription of *nptII*
12. Intervening sequence	Synthetic		3,115 – 3,122	8	Sequence used for DNA cloning
13. Native *Rpi-vnt1* gene promoter (pVnt1)	*S. venturii*	FJ423044	3,123 – 3,831	709	Drives expression of the *Rpi-vnt1* gene. Comprised of a promoter region and a 5' untranslated region.
14. *Rpi-vnt1* gene coding sequence (Vnt1)	*S. venturii*	FJ423044	3,832 – 6,507	2,676	Expresses the VNT1 protein for foliar late blight protection

15. Native *Rpi-vnt1* gene terminator (tVnt1)	*S. venturii*	FJ423044	6,508 – 7,432	925	Terminates transcription of *Rpi-vnt1*
16. Intervening sequence	Synthetic		7,433 - 7,438	6	Sequence used for DNA cloning
17. Native *Rpi-mcq1* gene promoter (pMcq1)	*S. mochiquense*	GN043561	7,439 – 9,496	2,058	Drives expression of the *Rpi-mcq1* gene. Comprised of a promoter region and a 5' untranslated region.
18. Native *Rpi-mcq1* gene coding sequence (Mcq1)	*S. mochiquense*	GN043561	9,497 – 12,082	2,586	Expresses the MCQ1 protein for foliar late blight protection
19. Native Rpi-mcq1 gene terminator	*S. mochiquense*	GN043561	12,083 – 13,226	1144	Terminates transcription of *Rpi-mcq1*
20. Intervening sequence	Synthetic		13,227 – 13,258	32	Sequence used for DNA cloning
21. Native *Rpi-blb2* gene terminator (tBlb2)	*S. bulbocastanum*	DQ122125	13,259 – 15,788	2,530	Terminates transcription of *Rpi-blb2*
22. *Rpi-blb2* gene coding sequence (Blb2)	*S. bulbocastanum*	DQ122125	15,789 – 19,678	3,890	Expresses the BLB2 protein for foliar late blight protection
23. Native *Rpi-blb2* gene promoter (pBlb2)	*S. bulbocastanum*	DQ122125rpi	19,679 – 21,208	1,530	Drives expression of the *Rpi-blb2* gene. Comprised of a promoter region and a 5' untranslated region.
24. Intervening sequence	Synthetic		21,209 – 21,291	83	Sequence used for DNA cloning
25. RB site	*A. tumefaciens* Ti-plasmid		21,292 – 21,316	25	Primary cleavage releases ssDNA T-DNA from pSIM4392

S2. Table 2. Assay optimization and quality testing of the Regions of Interest (ROIs). NTC = negative control, Tm = Temperature, ND = None Detected, QC = Quality control

Name	SEQUENCE	Region	Tm	Efficiency (%)	Background (NTC)	Status
_ROI1_8F	TGCGTTTGAATGTTTGCGGAA	Rpi-mcq1	79.0	92.8	ND	QC passed
_ROI1_8R	GTCTACCTCATCGCCGACAA	Rpi-mcq1				
_ROI2_9F	TCCTTTTGGTGTTTGGGAGTCT	Rpi-vnt1	76.0	97.0	ND	QC passed
_ROI2_9R	AGGAGGCATGATTCGGCAAA	Rpi-vnt1				

S2. Table 3. Links for Software, Tools, and Resources.

Software/Tools/Resource	Link
bedtools	https://github.com/arq5x/bedtools2
BLAST database	https://blast.ncbi.nlm.nih.gov/Blast.cgi
Guppy	https://github.com/timkahlke/LongRead_tutorials
Integrative Genomics Viewer (IGV)	https://github.com/igvteam/igv
minimap2	https://lh3.github.io/minimap2
samtools	https://github.com/samtools/samtools
seqkit	https://github.com/shenwei356/seqkit
Solanum tuberosum DM1-3 PGSC v4.04 pseudomolecules	http://spuddb.uga.edu/index.shtml

S2. Table 4. Primers used to confirm the junction sequence between inserted gene sequences and 1000bp of flanking sequence. UB015, UB255, UG234 and UG265 are abbreviated versions of DIA_MSU_UB015, DIA_MSU_UB255, GRA_MSU_UG234, and GRA_MSU_UG265 respectively.

Primer	Location	Sequence	Annealing Temp.	Product size (bp)
**UB015_FWD_RB**	Chromosomal	ACGATCCAATCTTCAC GTTAGC		
**UB015_REV_RB**	Right border T-DNA region	CTCTCTTCTTCTCCTTC AATCAG	50	1100
**UB015_FWD_RB2**	Chromosomal	ATCCACGTACTTGCCG ATTTG		

**UB015_REV_RB2**	Chromosomal	CAATAGTGAGACAGGT AATAG	50	700
**UB015_FWD_LB**	Chromosomal	TCCAGTTTAGACTTTGC ATG		
**UB015_REV_LB**	T-DNA 5'end of NPTII gene	CGTCCGTCCTCCAACTG CTTTC	59	600
**UB015_FWD_LB2**	Chromosomal	CATGGTACTCTCTTCGT TTT		
**UB015_REV_LB2**	Chromosomal	CGCATCTCTTCTTCACT CCATCTCC	55	1000
**UB255_FWD_RB**	Chromosomal	ATTCTCCCACCGTTTGT TTTCTTG		
**UB255_REV_RB**	Right border T-DNA region	CTCTCTTCTTCTCCTTC AATCAG	55	1500
**UB255_FWD_LB**	Chromosomal	GCTCTGTGTGCTAACCC TTCAA		
**UB255_REV_LB**	Left border T-DNA region	CTCAATCAATGTGGGA TCTCGATCGAGTTTCTC C	55	1500
**UG234_FWD_RB**	Chromosomal	CCTCTTTCTCTACAAGC CTA		
**UG234_REV_RB**	Right border T-DNA region	GCATAGATCGAGAGAA TCTGCAATGG	55	1400
**UG234_FWD_LB**	Chromosomal	AAATGAATCTCCCGTC CTCGAATC		
**UG234_REV_LB**	Left border T-DNA region upstream	CACACTCCCTGATTAGC TGATTC	55	1700
**UG265_FWD_RB**	Chromosomal	CATTTCCAGCACAAGG TCACATAC		
**UG265_FWD_RB**	Right border T-DNA region	GCATAGATCGAGAGAA TCTGCAATGG	55	1200
**UG265_FWD_LB**	Chromosomal	GAAGCCTGACTCCGCC AATG		
**UG265_REV_LB**	Left border T-DNA region upstream	GACGAAACCGCCTCAT AGGGT	55	1800
**UG265_FWD_LB2**	Chromosomal	GAAGCCTGACTCCGCC AATG		
**UG265_REV_LB2**	Chromosomal	GGACGGAAGGAGTAAC AAACT	55	850

S2. Table 5. Primers used to analyze the events at the insertion location sites and flanking regions in their non-transgenic potato variety. The insert location for event UG234 could not to be determined due to homologous regions found in the flanking regions of the T-DNA insert. UB015, UB255, UG234 and UG265 are abbreviated versions of DIA_MSU_UB015, DIA_MSU_UB255, GRA_MSU_UG234, and GRA_MSU_UG265 respectively.

Primer	Location of primer insertion of T-DNA	Sequence	Annealing Temp.	Expected Product size (bp)
**UB015_FWD**	Chr 1 at 66240633 Upstream	GAGGGAGAGGGTCAGGG ACCTAAAAGT		
**UB015_REV**	Downstream	GTACGTGGATTTGGCAA AACCAGTTAGAG	60	1435 bp
**UB255_FWD**	Chr 11 at 27630548 Upstream	GAGCTACCCAACACCTC ATTATTC		
**UB255_REV**	Downstream	AAGAGGAGGGACAAGTG TATTC	60	1296 bp
**UG234_FWD**	Not able to be determined			
**UG234_REV**	Not able to be determined			
**UG265_FWD**	Chr 5 at 1082057 Upstream	AGTTTGTTACTCCTTCCG TCC		
**UG265_FWD**	Chr 5 at 1082057 Downstream	AGGGTTTGAGGAGAGAG TCTTAGG	55	3656 bp

S2. Table 6. Primers used to confirm T-DNA sequence stability across clonal cycles. UB015, UB255, UG234 and UG265 are abbreviated versions of DIA_MSU_UB015, DIA_MSU_UB255, GRA_MSU_UG234, and GRA_MSU_UG265 respectively.

Primer	Location in pSIM4392	Sequence	Annealing Temp.	Product size (bp)
**VNT1 FWD**	5851	ATGTTACTGTGTCTCTTTTGC		
**VNT1 REV**	6222	GCCGAGAAAGTATCCATCAT	50	371
**p4274F1(vnt1 terminator)**	7064	GCATTTGGTGATCTCGGCACT		
**p4274R1(mcq1 promoter)**	7603	TTTGTTAAGTCTTTGGGGTATATT	50	512
**UB015_FWD_RB**	20990 Right Border within *Rpi-blb2*	CTCTCTTCTTCTCCTTCAATCAG		
**UB015_REV_RB**	Chromosomal	AACAAATCGGCAAGTACGTGG	55	849
**UB255_FWD_RB**	20990 Right Border within *Rpi-blb2*	CTCTCTTCTTCTCCTTCAATCAG		
**UB255_REV_RB**	Chromosomal	ATTGGCCGTTTGAACCTCATTGC	55	675
**UG234_FWD_RB**	21121 Right Border within *Rpi-blb2*	GCATAGATCGAGAGAATCTGCAAT GG		
**UG234_REV_RB**	Chromosomal	CCTCTTTCTCTACAAGCCTA	50	1400

**UG265_FWD_RB**	20990 Right Border Within *Rpi-blb2*	CTCTCTTCTTCTCCTTCAATCAG		
**UG265_REV_RB**	Chromosomal	TGACAGCCCAACAAGAAGAAC	55	636
**UB015_FWD_LB**	1941 Left Border	CGTCCGTCCTCCAACTGCTTTC		
**UB015_REV_LB**	Chromosomal	TCCAGTTTAGACTTTGCATG	55	600
**UB255_FWD_LB**	271 Left Border	CTCAATCAATGTGGGATCTCGATC GAGTTTCTCC		
**UB255_REV_LB**	Chromosomal	GCTCTGTGTGCTAACCCTTCAA	55	1500
**UG234_FWD_LB**	1719 Left Border	CACACTCCCTGATTAGCTGATTC		
**UG234_REV_LB**	Chromosomal	CACCCACATCACAAGATCAT	55	1438
**UG265_FWD_LB**	1141 Left Border	GATGGCATGGCCGACTCTAG		
**UG265_REV_LB**	Chromosomal	AGTTTGTTACTCCTTCCGTCC	55	515

S2. Table 7. ddPCR NPTII Copy Number Analysis. The results show that there are single *nptII* gene insertions in the T-DNA region, in the four events tested, and no insertions in the non-transgenic control varieties.

Sample Name	ng/ul DNA	Ratio (nptII:StAAP2)
**Diamant**	80.2	0.2
**DIA_MSU_UB015**	21.1	1.2
**DIA_MSU_UB255**	29.6	1.2
**Granola**	44.6	0.2
**GRA_MSU_UG234**	18.2	1.2
**GRA_MSU_UG265**	18.1	1.2

S2. Table 8. Overview of the samples, assays, DNA yields, and enrichment after the dMDA reactions.

Sample	dPCR Assay	DNA concentration (ng/µL)	Volume (µL)	DNA amount (ng)	qPCR assay	Fold enrichment
DIA_MSU_UB015	R0 12 8	61	40	2440	R012 9	<100
DIA_MSU_UB255	R0 12 8	39	40	1560	R012 9	<100
GRA MSU UG234	R0 12 8	43	40	1720	R012 9	114
GRA MSU UG265	R0 12 8	30	40	1200	R012_9	161

S2. Table 9. Overview of ONT sequencing results. The barcode used for each sample and the raw amount of data produced.

Sample	Droplet ID	dPCR assay	Barcode	Amount of raw data (Gb)
DIA_MSU_UB015	20220228-d03	ROI2_8	2	1.3
DIA_MSU_UB255	20220228-d04	ROI2_8	3	1.2
GRA MSU UG234	20220228-d05	ROI2_8	4	1.4
GRA MSU UG265	20220228-d06	ROI2_8	5	1.2

S2. Table 10. Flanking sequences of the T-DNA Inserts for 3-R-Gene LB events obtained by Xdrop®/Nanopore sequencing and confirmed by Sanger sequencing.

**Event**	**Junction sequence adjacent to left border region: 1kb flanking and 500bp of T-DNA**	**Junction sequence adjacent to right border region: 1kb flanking and 500bp of T-DNA**
DIA_MSU_UB015	**1KB FLANKING**<br>TCTCCATAGCCTCACCCACCACCCCA<br>TATCTCTACCCCTACTCCTACTTCCCA<br>TACACTATAAAATCTTATTAGATAAA<br>TTTAAAGTCTCTTATTAAAGTTATGC<br>TACCAATTTAGTTGAGATACCCCTAC<br>TAATTACATTATTAATTACTGTATAG<br>CTAATCTATCTAGCACCAATCCTAAT<br>TCCTAAATATTAATAATATCTTTAAA<br>TTAAACTACCCTCAAATTTCATATGT<br>AAAACACATATCTTGTATTAATTGAT<br>TCTATCCAAATACTTGTGCTGACAAG<br>TTTTTATCTCTGTCGTGTTAATGATTT<br>ATTTTTCTCCCCTTTTTTAGGAGGAA<br>AAAAAGCTGATTTTAAATGATTTTGT<br>CAATGCTTAATATTTATATAGCACTA<br>ATAATTATGAGGGAGAGGGTCAGGG<br>ACCTAAAAGTCAACTATAAAACCAA<br>ACAATAAAATTCCTATCCTAATTTTA<br>AAAAATAAGTTAGGTTGATTTTAAAA<br>AAAAAAGGAATTTATGGTTAATTAA<br>ACATAAACTGGCCCTAAGTATATTTT<br>CGGTCGTTTCATATTACTTGATTCCA<br>ATATATATATATATATATATATATTTT<br>AATTGAGAAAATCAAGAGAATTTTAT<br>CTTTTTTTTTTCTTTATACTCTTAGTA<br>ATCACTAATCAGTATCAAATAACTAA<br>ATAAAACGATAGTAGTCAAATTTAG<br>AATATTAATGTACCATTATTCATGTT<br>AGTTTAGTTAAATACACCCCCAAAAC<br>TCTCTATTAACTTTTACTTGTCCAGTT	**1KB FLANKING**<br>INSERTIONAGTCAAACTCTCACCATTTATCA<br>GAGAGAATTTTAAATATTGAACTTTACTATC<br>TAGCTATCTACACTTACAAATATGGATTAAT<br>TTCATTTGTACTAGGTGAAATATTAATTCGA<br>AGAGTATTTTAAGTGAAATAATAATTTATC<br>GACCAATCTTTTAATTTTCAAAAATATTTTC<br>AATCAACATAATCTTCAACACAAAGGTTTA<br>GCTTTATAAAAATCCTAAAAAGAGAGCATA<br>TAAATTGCCACTTTTTAGAACTTTTACCTTT<br>AGTCAATAACTTTTTTTTATCAACATAATAT<br>AATCTTTTTGTTTTTTTTTCTCTTAAGTTACA<br>AAGATGAACTTTTTCCATTCCAAGAAAGTT<br>CGTAGATAAATCCTAAGAAAAAAAGTCATC<br>AATTATGGGAAGTCAAAAAATTGATTTTTTT<br>CCTTATTTTGACCGAAGGGATTATTTTTAAA<br>TAATATGATCGAAAACATGTGATTTAATTA<br>AAAAATACTTTATTTCTCTAACTGGTTTTGC<br>CAAATCCACGTACTTGCCGATTTGTTTGATA<br>AGCTGTTTTTGAGTCAACTTTCTTTATTATT<br>ACTACTTTTTATTTTTATTTTTTTGGTATTGC<br>AGCCATAAAATGGGAAAACAGAAAAAGAG<br>AAACTTTCTGTTCCCACAAACTTTGGCTATA<br>TTTAAATATTGTTTGGCTGAAATAATAAAAT<br>TAAAATTAAATTAGAAGGACCATCATAGAG<br>TTTTAGCTTTTGACATCTTTTTCTTTTCAATT<br>TTCTATTAAAAAAATCTTTCAGTTTGCTAAC<br>GTGAAGATTGGATCGTATAATAAACACGTA<br>AAAACTGTCTATAAAAATATATATATATAT<br>ATATATATAATATCCACATACCTTATGATTA

	TAGACTTTGCATGCTTAAGTAAAAGT TTTTTTTTGAGAGAGTAAAATAGATT TTTTTTTGTGTTTTTTTATATGTTAAA AATGACAAGTAAAACTAATTTTTTTT TTTAAATAGTTGATAAAAACGAACA AAGAGAGGAAAACTTTCTTGAGAAT TGTGTTAAGTCAATATGAGTCAAGTA ATATAAAACGAAGAGAGTACCATGT AATTTGTCCAAACINSERTION<br><br>**500KB T-DNA**<br><br>INSERTIONATGAAAAAGCCCTAGAAT TTGTGTTTTTTCGTCGGTTTGATTCTG AAGGCCTAAAATTTGAGTTTCTCCGG CTGTTTTGATGAAAAAGCCCTAAATT TGAGTTTCTCCGGCTGTTTTGATGAA AAAGCCCTAAATTTGAGTTTTTTCCC CGTGTTTTAGATTGTTTGGTTTTAATT CTCGAATCAGCTAATCAGGGAGTGTG AAAAGCCCTAAAATTTGAGTTTTTTT CGTTGTTCTGATTGTTGTTTTTATGAA TTTGCAGATGCAGATCTTTGTGAAAA CTCTCACCGGAAAGACTATCACCCTA GAGGTGGAAAGTTCTGATACAATCG ACAACGTTAAGGCTAAGATCCAGGA TAAGGAAGGAATTCCCCCGGATCAG CAAAGGCTTATCTTCGCCGGAAAGCA GTTGGAGGACGGACGTACTCTAGCTG ATTACAACATCCAGAAGGAGTCTACC CTCCATTTGGTGCTCCGTCTACGTGG AGGTGGATCTATGA	TTTTTTTTTGTTGTTTTTGACGCGGGGAAAT TATTATTTTTTAATTATTTTATAGAAAGAA<br><br>**500KB T-DNA**<br><br>AGCAGTTAGTTAAATGCACTGCAGTGCACT AACAGAAAATGTACACCTATCAGTCTAGTT GTTAGAGTCAGTTAAATTAACTGCTTCAGA TTAGAATATAAATAGAGATATATAGATTGT AATAGTATAGTTCTTCTCATTATTTTTTTCAT TCATTTTGGTTCATGAGTTCAATATAGAATT TTTCTCTCTTCTTCTCCTTCAATCAGTCTTTC TAAAATAGTTAATTCTCTCCATTGTTTAGTT AGAATTCACTGAAATTACATGGTATCAGAG CATTTGAAGCAGATCTAGGGTTTTATCTCGG GATTTTTTGAGCATAGATCGAGAGAATCTG CAATGGCGATTGAAGAGGAATCAGTGGATG AAATTCATGGTAGAACTGGAGGAGCTGTAC CGAGGGGAGGTTCGGTGATTCTAGATCCTC TAGAGTCGACCTCGAGGGGGGGCCCACTCG AGATGGTGACCGAGCTCAATTAAACTATCA GTGTTTGAAGTCAINSERTION
DIA_MSU_UB255	**1KB FLANKING**<br><br>TTTTTTTCATAATAAAAATAAATAAA TTGGTTCAAATTTCAAGAGAGTTATT AATTTTTTTTTTCCAATTTTGCCCCGT CATTTTTATTTTAGGTGATATTTCAAG AGAGTTAATTGTTCATATTTATGATT TAACTTTGAATTATTTATATTTTCAAG AATAGTTTGAGAATAACTAAAGGGC GAAAATAGAAAGTGGTATTTATTTTA TGTCCTGAACTTTTTTTCTAAATAAAT ATGCATTACTTTAACAATTTACTTATT TTGAAGCGGAGAAAATAAGCACGAA ATCTTGTATCTAGTACCAGAGAGAAA AATATAAATGTTGTTCACATTAATAA AATTTCTGAAAATCCACATTTCACC ACCTGATACACAATGTGTCTATCTAT GCGAACTTTTTTGTCAACACCAAAAT CTACTTTGACCTTTTGCAATTGAGCT ACCCAACACCTCATTATTCTAAATTT AAAGTTTGTGTATGATACTTAATACA TATTATTTATGTTATTAGTTAATACTT TAAGAATCCATATATATGTACGTGGA	**1KB FLANKING**<br><br>INSERTIONCTAGTTTCCTCCAAACTTCACTT CCATATTAATTTTTTGAGTGACCAAATTTAA AATGGAAGATGATCAAGAAAAGTAAGAAT AATATCGACATGTATAGAGGAGTAAGGAAG AGAAAATGGGGGAAATGGGTATCTGAGAT ACGCGAACCAGGAAAGAAAACTCGAATAT GGTTGGGGAGTTATGAGACAGCAGAAATGG CTGCTGCAGCTTATGATGTTGCTGCATTTCA TCTAAAAGGTGAGAGACCAAACTTAAGGCT CAATTTCCCTGAATTAATACATACATTCCCA AAACCCTCAAGTTCAAGACCTGAAGATGTA CAAATGGCAGCTCATGAAGCGGCAATGAGG TTCAAACGGCCAATTGATGATCATCCAGAC AACTGTGGTGCAGGCCCGGTGAGAGTAGGT CTCTCACCGAGTCAAATTCAGGCGATTAAT GAATCCCCGTTGGACTCCCCAAAAATGTGG ATGGAGTTTGCCGGGGCTTTATTACCTGTTA GAGAATACACTTGTCCCTCCTCTTCTTTTGA GGATCATCAGTGGGATGAAATCCAATACCA TCATGATTCCATTTGGGATTTTTAATTTCCA TATATATGCCTCAACCCCTTTAATTAGTATA

	CCACGAGGTCCATATATCACATTATT CAAATTAAAAAATGGATAAAGAAAA AAAAATAAGTATATTAACACGTCGC ATCATCATATATACTCCCTATGTCCC TAATTACTTGTCTACTTTTGAATTGTC ATACCTATTAAGAAAATAATGATTGA CATAACGAGTTTATCATTTTACCCCT ATTAATTATGAAGTAGATGTTGGGTT CTGTGAATTCAGATAGTTCTTTTCTTT TTCTTTTCATCTTAATTATTTCTGATT TGCTAATGTGAATAGAGGAGACAAC AATAGATGAATTAAATACTTAAGCTT TTTATGAAGTTCTACGTTTTCAAATT AATTAATTAAGGATATAATAGGTAA AAAAAAATTGTCATTTCTTGA<br>INSERTION<br><br>**500KB T-DNA**<br><br>INSERTION<br>TAGAAATTTTATTGATAGAAGTATTT TACAAATACAAATACATACTAAGGG TTTCTTATATGCTCAACACATGAGCG AAACCCTATAGGAACCCTAATTCCCT TATCTGGGAACTACTCACACATTATT ATGGAGAAACTCGATCGAGATCCCA CATTGATTGAGTTTTATATGCAATAT AGTAATAATAATAATATTTCTTATAA AGCAAGAGGTCAATTTTTTTTTAATT ATACCAACGTCACTAAATTATATTTG ATAATGTAAAACAATTCAATTTTACT TAAATATCATGAAATAAACTATTTTT ATAACCAAATTACTAAATTTTTCCAA TAAAAAAAAGTCATTAAGAAGACAT AAAATAAATTTGAGTAAAAAGAGTG AAGTCGACTGACTTTTTTTTTTTTTAT CATAAGAAAATAAATTATTAACTTTA ACCTAATAAAACACTAATATAATTTC ATGGAATCTAATACTTACCTCTTAGA AATAAGAAA	TTTTAGACAAAATTATTGGGGGCGAACAAA AGGAATTTTATGCCATTATATTCTTTTAAAT TTTTGTTTAATAGAGATTCTTCGTTAGAATA AGTGTTACTAATCTCGAGACAAGTACGTCC ATGAAGATGGTCAAAAATAGAAGATAATG GTGGAGCCTTCAATTTTATTTTGCTTTTTCT ATAGCCTTTAATATTTCTTTCCCTTGTAAAT CTATATCTATTTACGAGATTTTGCGACATTA CTTAGTTTATGTCCCAAAAAATCACATAGA CAAGTATTTCACTTGAGATGGTAAATTTTTC ACAATTCTACAATTTTACAAGGGTAGTATA TTGTATTAAATGATATATGGACATCGTACA AAATAACT<br><br>**500KB T-DNA**<br><br>ATGTTAAGCAGTTAGTTAAATGCACTGCAG TGCACTAACAGAAAATGTACACCTATCAGT CTAGTTGTTAGAGTCAGTTAAATTAACTGCT TCAGATTAGAATATAAATAGAGATATATAG ATTGTAATAGTATAGTTCTTCTCATTATTTT TTTCATTCATTTTGGTTCATGAGTTCAATAT AGAATTTTTCTCTCTTCTTCTCCTTCAATCA GTCTTTCTAAAATAGTTAATTCTCTCCATTG TTTAGTTAGAATTCACTGAAATTACATGGTA TCAGAGCATTTGAAGCAGATCTAGGGTTTT ATCTCGGGATTTTTTGAGCATAGATCGAGA GAATCTGCAATGGCGATTGAAGAGGAATCA GTGGATGAAATTCATGGTAGAACTGGAGGA GCTGTACCGAGGGGAGGTTCGGTGATTCTA GATCCTCTAGAGTCGACCTCGAGGGGGGGC CCACTCGAGATGGTGACCGAGCTCAATTAA ACTATCAGTGTTTG INSERTION
GRA_MSU_UG234	**1KB FLANKING**<br><br>INSERTION<br>CTTTTCCCCAAAAGTTAGGGTTTTAC TCCAAGTCATGGATTCCTTTTTTAAA ATGATTCTATTGGTTTAATTACTTGAT ATTATGATTGAATTGATGATTTATTA TTGTTTTATGATGAAATCTCCTAAAA ATCCATGAATCCCCAATATTCCCAAA TTATGATCTTGTGATGTGGGTGAATT GTTTGACATTATTCTTATAGTTATTAT ATTAATTGAATCATGTTATTATCCAT GTCTCATCTAGTAATTCTAGATGTGT TGATTGAATGCGATATATGGTCCTTG AAGGGCAAAGCATGAGAATTATGCA TGATTGTGAGGTTTATGTATATGCTT TTATAAAACTTTGAGAGAAAGGTGA	**1KB FLANKING**<br><br>INSERTION<br>TCATGACTTGGAGTAAAACCCTAATTCTTG GGGTGTGATCATGTGATTATTAGTTCAAATT AGCTTATGGTTATTGTTTTTTACTAATTTAA ATGATTAATTAAAGTAGGTTTAATTAATTA GCTATGAATTTATGTAAATTCAATTATATGT TAGTGTTTTAGGCTTGTAAAGAAAGAGG<br><br>(1KB WAS NOT ATTAINABLE DUE TO HOMOLOGOUS REGIONS.)<br><br>**500KB T-DNA**<br><br>TCATGTAAAAAATATATACTAAAAATAACT TAATTTTATTTTAGGTATTTTTTATTTTAAT

	ATATTATTATGATCTAGTTGTTGTGTT GTTGGATGATTATCCTCATGTACACA CATATGCATGAACATGATATCTTCTG AAAGGTTTGTCACATGCAGTGATTCT AAAGTTGGATGACTGGTCTCACCTAT TGAATCTATTAGATTCAATATGACTT ATGTTGTATGAGTGTCAAGATATTTC TTCCATAATTATGAACTTTAAATAAG AGACTTCGTATGAACTCCGTCGGTAT TAATGCTTAGCACTGAGGTGATAAGT AATATGACCTGGAAATTCTCCTCGTT AGTAGAGGTGGGATTTTTTTATTAGC AATCCCCTTTATCCCATAAA<br><br>**500KB T-DNA**<br><br>TATACTAAAAATAACTTAATTTTATT TTAGGTATTTTTTTATTTTAATATATG CTCTAATATAGCGCATTTCAAGTTTA TCATGAACATGACCTTAAATATAAGA GTTTAAATATTAGCAAATTAGGATAT AATACTACCTTGATAATCGCACTTAA ATCTTCTTTCTTATATTATGTATTGCT CTTTTTTCATATTATTTTGATATTGTT ATTGACCTCATATTGATATTTGTTATT TAAATTTGATTGAAAATATTTAATGT AAATTGAGGGTCTATTAGAAACACTT TTTCTTATTTCTAAGAGGTAAGTATT AGATTCCATGAAATTATATTAGTGTT TTATTAGGTTAAAGTTAATAATTTAT TTTCTTATGATAAAAAAAAAAAAGTC AGTCGACTTCACTCTTTTTACTCAAA TTTATTTTATGTCTTCTTAATGACT INSERTION	ATATGCTCTAATATAGCGCATTTCAAGTTTA TCATGAACATGACCTTAAATATAAGAGTTT AAATATTAGCAAATTAGGATATAATACTAC CTTGATAATCGCACTTAAATCTTCTTTCTTA TATTATGTATTGCTCTTTTTTCATATTATTTT GATATTGTTATTGACCTCATATTGATATTTG TTATTTAAATTTGATTGAAAATATTTAATGT AAATTGAGGGTCTATTAGAAACACTTTTTCT TATTTCTAAGAGGTAAGTATTAGATTCCATG AAATTATATTAGTGTTTTATTAGGTTAAAGT TAATAATTTATTTTCTTATGATAAAAAAAAA AAAGTCAGTCGACTTCACTCTTTTTACTCAA ATTTATTTTATGTCTTCTTAATGACTTTTTTT TATTGGA INSERTION
